# Osteocrin ameliorates adriamycin nephropathy via p38 mitogen-activated protein kinase inhibition

**DOI:** 10.1038/s41598-021-01095-8

**Published:** 2021-11-08

**Authors:** Takaya Handa, Keita P. Mori, Akira Ishii, Shoko Ohno, Yugo Kanai, Haruko Watanabe-Takano, Akihiro Yasoda, Takashige Kuwabara, Nobuyuki Takahashi, Naoki Mochizuki, Masashi Mukoyama, Motoko Yanagita, Hideki Yokoi

**Affiliations:** 1 Department of Nephrology, Graduate School of Medicine, Kyoto University, 54 Shogoin Kawahara-cho, Sakyo-ku, Kyoto, Kyoto 6068507 Japan; 2 Department of Nephrology and Dialysis, Tazuke Kofukai Medical Research Institute, Kitano Hospital, Osaka, Japan; 3 TMK Project, Medical Innovation Center, Graduate School of Medicine, Kyoto University, Kyoto, Japan; 4 Department of Diabetes Mellitus and Endocrinology, Osaka Red Cross Hospital, Osaka, Japan; 5 Department of Cell Biology, National Cerebral and Cardiovascular Center, Research Institute, Suita, Japan; 6 Clinical Research Center, National Hospital Organization Kyoto Medical Center, Kyoto, Japan; 7 Department of Nephrology, Kumamoto University Graduate School of Medical Sciences, Kumamoto, Japan; 8 Department of Clinical Pharmacology and Therapeutics, Tohoku University Graduate School of Pharmaceutical Sciences and Medicine, Sendai, Japan; 9 Institute for the Advanced Study of Human Biology (ASHBi), Kyoto University, Kyoto, Japan

**Keywords:** Nephrology, Kidney diseases

## Abstract

Natriuretic peptides exert multiple effects by binding to natriuretic peptide receptors (NPRs). Osteocrin (OSTN) binds with high affinity to NPR-C, a clearance receptor for natriuretic peptides, and inhibits degradation of natriuretic peptides and consequently enhances guanylyl cyclase-A (GC-A/NPR1) signaling. However, the roles of OSTN in the kidney have not been well clarified. Adriamycin (ADR) nephropathy in wild-type mice showed albuminuria, glomerular basement membrane changes, increased podocyte injuries, infiltration of macrophages, and p38 mitogen-activated protein kinase (MAPK) activation. All these phenotypes were improved in OSTN- transgenic (Tg) mice and NPR3 knockout (KO) mice, with no further improvement in OSTN-Tg/NPR3 KO double mutant mice, indicating that OSTN works through NPR3. On the contrary, OSTN KO mice increased urinary albumin levels, and pharmacological blockade of p38 MAPK in OSTN KO mice ameliorated ADR nephropathy. In vitro, combination treatment with ANP and OSTN, or FR167653, p38 MAPK inhibitor, reduced *Ccl2* and *Des* mRNA expression in murine podocytes (MPC5). OSTN increased intracellular cyclic guanosine monophosphate (cGMP) in MPC5 through GC-A. We have elucidated that circulating OSTN improves ADR nephropathy by enhancing GC-A signaling and consequently suppressing p38 MAPK activation. These results suggest that OSTN could be a promising therapeutic agent for podocyte injury.

## Introduction

Adriamycin (ADR) is a well-known toxic agent that causes podocyte injury and foot process effacement, following renal injury in rodents. The mechanism of ADR is DNA intercalation and inhibition of macromolecular biosynthesis^1^. ADR-induced nephropathy is an animal model of nephrotic syndrome. ADR-administered mice show reduced glomerular cells and mesangial expansion in the kidney, and electron microscopy shows wide effacements of foot process and thickening of the basement membrane^2^. ADR-administered mice also exhibit, at early stages, accumulation of macrophage which predicts subsequent disease progression^3,4^, and upregulation of desmin, a podocyte injury marker^5^. The phenotype of ADR-induced nephropathy depends on the animal background. Male BALB/c mice on 129SvJ are susceptible to ADR injection whereas C57BL6/J mice are resistant to ADR injection^2^. Here, we treated mice with ADR to reveal protective roles of natriuretic peptides (NPs) in podocytes.

NPs are hormones that reduce blood pressure, inhibit ventricular hypertrophy, and promote bone growth. The mammalian members of NPs are atrial natriuretic peptide (ANP), brain natriuretic peptide (BNP), C-type natriuretic peptide (CNP), and osteocrin (OSTN)^6,7^. Three natriuretic peptide receptors (NPRs) have been reported: NPR-1/NPR-A/GC-A, NPR2/NPR-B/GC-B and NPR3/NPR-C. GC-A and GC-B contain guanylyl cyclase domain that can synthesize cyclic guanosine monophosphate (cGMP) by ANP and BNP stimulation, whereas NPR-C, coded by the *NPR3* gene, lacks a guanylyl cyclase domain and works as natriuretic peptide clearance reporter^7,8^. ANP, BNP, and CNP are degraded by binding to NPR-C through internalization followed by lysosomal degradation. The affinity of NPR-C for NPs is ANP > CNP > BNP in both humans and rats^9,10^, which may be related to the much shorter half-life of plasma ANP than that of BNP. Inactivation of NPR-C has been reported to increase the half-life of ANP in circulation by two-thirds in mice, and to show lower blood pressure^11^. Inactivation of NPR-C is also expected to block the degradation of ANP and subsequently to promote GC-A signaling.

GC-A is expressed in the kidney^6,12^, especially in podocytes as well as in collecting ducts and distal tubules^13^. Since podocyte-specific GC-A knockout (KO) mice with aldosterone, high salt, and uninephrectomy exhibit podocyte injury with augmented phosphorylation of p38 mitogen-activated protein kinase (MAPK) and its inhibition ameliorates glomerular injury, p38 MAPK is presumed to mediate downstream signaling of GC-A^14^. NPR-C is also expressed in the kidney^12,15^, however, its role and the cell types expressing it remain unknown.

OSTN is a relatively newly identified peptide in osteoblasts acting a soluble osteoblast regulator^16^, and is also known as musclin, given that another group identified *Ostn* mRNA expression in skeletal muscles^17^. It binds with high affinity to NP clearance receptor, NPR-C^18^, and inhibits NP degradation and increases the circulating levels of NPs, which bind to GC-A and activate G-protein coupled receptors and peroxisome proliferator-activated receptor gamma coactivator 1-α (PGC-1α) pathways^19–21^. OSTN has multiple effects on various organs. OSTN has been shown to strongly affect glucose metabolism in animal models via the inhibition of phosphatidylinositol 3-kinase (PI3K) and Akt^17,22^. OSTN also prevents the worsening of congestive heart failure after myocardial infarction^20^ and doxorubicin-induced cardiotoxicity^23^. OSTN-transgenic mice with elevated circulating levels of OSTN showed skeletal overgrowth^24^, while OSTN knockout mice exhibited shortening of some long bones^25^. ANP^6^ and BNP^26–28^ play protective roles in the kidney by directly binding to NPRs in the kidney, but the role of OSTN in the kidney has not yet been well elucidated.

In the present study, we investigated the significance of excess circulating OSTN in ADR nephropathy, OSTN deficiency in ADR nephropathy, and the mechanism of OSTN using NPR-C KO mice, p38 MAPK inhibitors, and cultured podocytes.

## Results

### Overexpression of OSTN in circulation ameliorates adriamycin nephropathy

**Figure 1:**
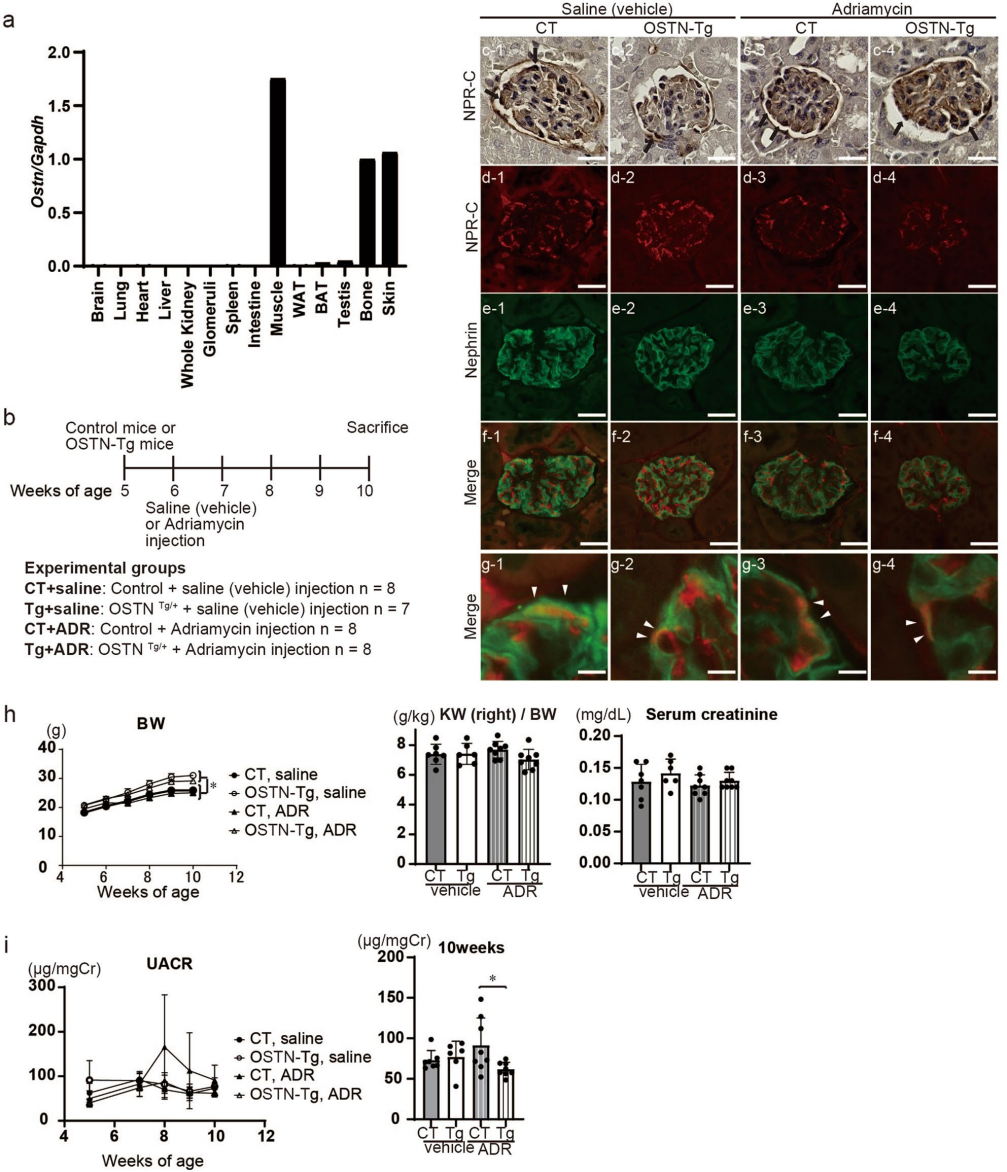
Urinary albumin levels were lower in OSTN-Tg mice than in control mice under ADR nephropathy. (**a**) Expression of *Ostn* mRNA in various tissues of wild-type mice. Skin (skin of the auricular region). (**b**) Schematic of the experimental protocol. OSTN-Tg or control mice were injected intravenously with saline or ADR (8 mg/kg) at 6 weeks of age, and sacrificed at 10 weeks of age. (**c**) Immunohistochemical study for NPR-C. Bars, 20 μm. (**d**–**g**) Immunofluorescent studies for NPR-C (**d**; red), nephrin (**e**; green), and merged images (**f**,**g**) in four groups. (**d**–**f**) Bars 20 μm, (**g**) Bars 4 μm. (**h**) The BW at weeks 5, 6, 7, 8, 9 and 10 and right kidney weight, serum creatinine levels at weeks 10 (n = 7–8 per group). *P < 0.05, CT + vehicle vs. Tg + vehicle, or CT + ADR vs. OSTN KO + ADR. **P < 0.01, ***P < 0.001 by one-way ANOVA analysis. (**i**) Urinary albumin creatinine ratio (UACR) at weeks 5, 7, 8, 9 and 10 (n = 7–8 per group). Tg 61.9 ± 8.5 μg/mgCr vs. CT 91.5 ± 33.7 μg/mgCr. BW, body weight; CT, control mice; Tg, OSTN-Tg mice. Data are mean ± SD. *P < 0.05 by one-way ANOVA analysis.

To investigate the role of OSTN in podocyte injury, we used ADR nephropathy model, which presents with massive proteinuria similar to human minimal change nephropathy. We have previously reported that p38 MAPK plays essential roles in ADR nephropathy^29^, and that GC-A signaling pathway ameliorates podocyte injury and apoptosis via p38 MAPK inhibition^14^. It is reported that p38MAPK mediates upregulation of renal MCP1^30–33^, which is an important factor in glomerular injury^34,35^. We hypothesized that OSTN binds to NPR-C in the kidney, thereby enhancing GC-A signaling. We first examined mRNA expression of *Ostn*. Expression of *Ostn* in muscle, bone and skin (auricle) was more prominent than in brain, lung, liver, whole kidney, glomeruli, spleen, intestine, white adipose tissue (WAT), brown adipose tissue (BAT) and testis (Fig. 1a). To determine whether circulating OSTN plays a reno-protective role in ADR nephropathy, we used liver-specific driven human serum amyloid-P component (SAP) promoter-driven OSTN-transgenic (Tg) mice, which have plasma OSTN levels 2000 times higher than control (CT) mice^24^. We injected ADR or saline (vehicle) into CT or Tg mice at 6 weeks of age (Fig. 1b). We confirmed NPR-C colocalized with nephrin, indicating expressed by podocytes (Fig. 1c–g). The BW of Tg mice was significantly higher than that of CT mice regardless of ADR or vehicle injection (Fig. 1h), and these finding was consistent with previous studies and might be related to bone growth stimulated by OSTN^24^. There were no significant differences in kidney weight, serum creatinine levels (Fig. 1h) or systolic blood pressure (Fig. S1). The urinary albumin-to-creatinine ratio (UACR) peaked at 8 weeks of age in ADR-injected CT (ADR CT) mice. The UACR in ADR-injected Tg (ADR Tg) mice was significantly lower than that of ADR CT mice at 10 weeks of age (Fig. 1i).

**Figure 2:**
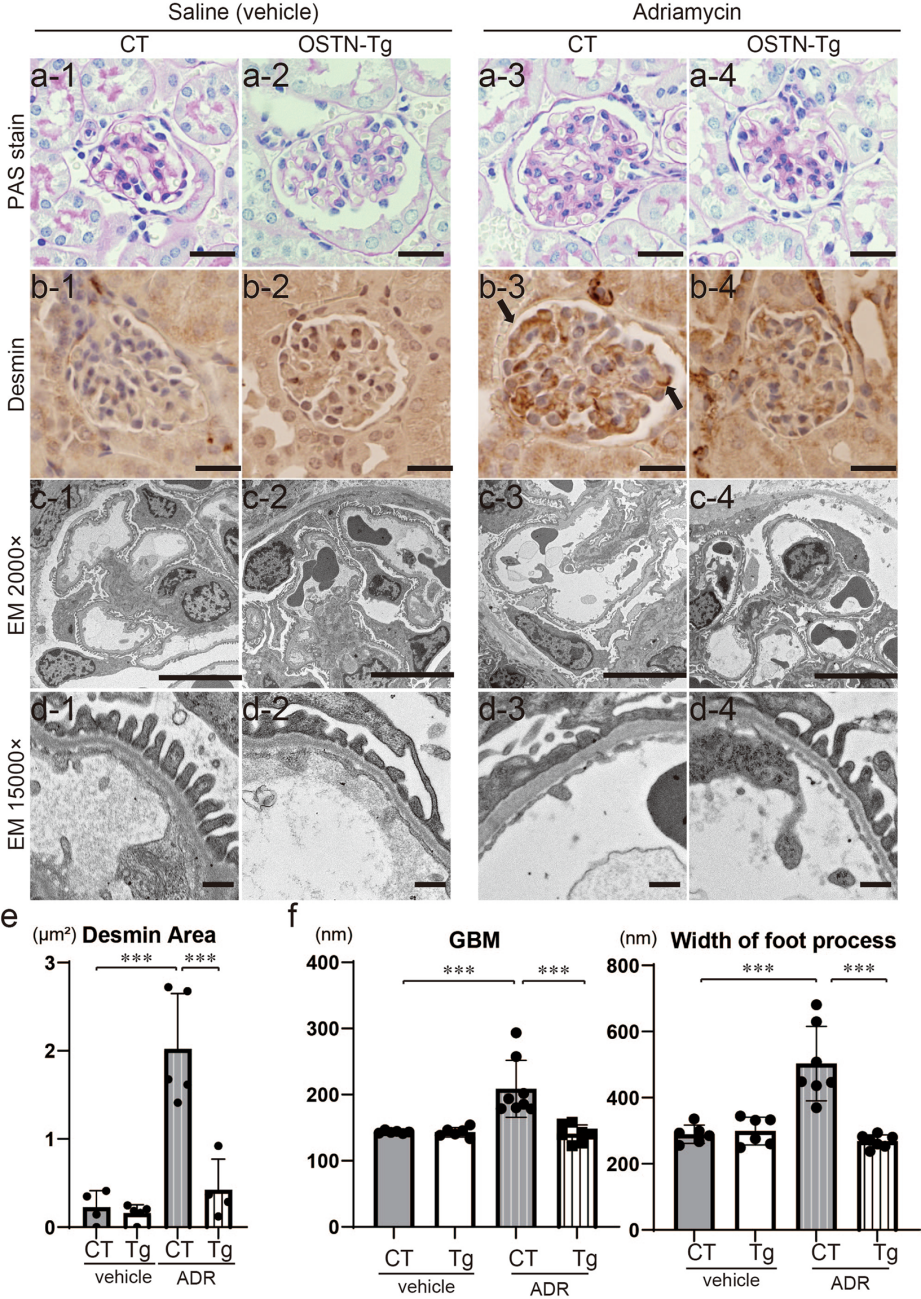
ADR-induced podocyte injury was ameliorated in OSTN-Tg mice. (**a**) Light microscopic analysis stained with PAS. Bars, 20 μm. (**b**) Immunohistochemical study for desmin (magnification, × 400). Bars, 20 μm. (**c**,**d**) Electron microscopic analysis with lower magnification (**c**; magnification, × 2000, Bars, 10 μm), and higher magnification (**d**; magnification, × 15,000, Bars, 500 nm). (**e**) Desmin-positive areas were analyzed. ADR Tg 0.42 ± 0.35 μm^2^ vs. ADR CT 2.02 ± 0.63 μm^2^. (**f**) Thickness of glomerular basement membrane (GBM) and width of foot process effacements were measured. GBM thickness: Tg 140.9 ± 13.2 nm vs. CT 208.7 ± 43.1 nm, *P* < 0.001; width of foot process: Tg 268.3 ± 19.3 nm vs. CT 502.7 ± 42.6 nm. *PAS* periodic acid-Schiff staining, *EM* electron microscopy, *CT* control mice, *Tg* OSTN-Tg mice. Data are mean ± SD. ***P < 0.001 by one-way ANOVA analysis.

The histological findings at 4 weeks after ADR induction are presented in Fig. 2. PAS stain showed no changes in glomerular findings between CT and Tg mice regardless of ADR or vehicle injection at 4 weeks after ADR administration (Fig. 2a) The positive area of desmin, a marker of podocyte injury, was significantly reduced in ADR Tg mice compared to that in ADR-injected CT mice (Fig. 2b,e). Foot process effacements observed in ADR CT mice were ameliorated in ADR Tg mice in electron microscopic findings (Fig. 2c,d). Glomerular basement membrane (GBM) thickness and width of foot processes were significantly mitigated in ADR Tg mice (Fig. 2c,d,f). These histological findings indicated that ADR-induced podocyte injury was improved in Tg mice.

**Figure 3:**
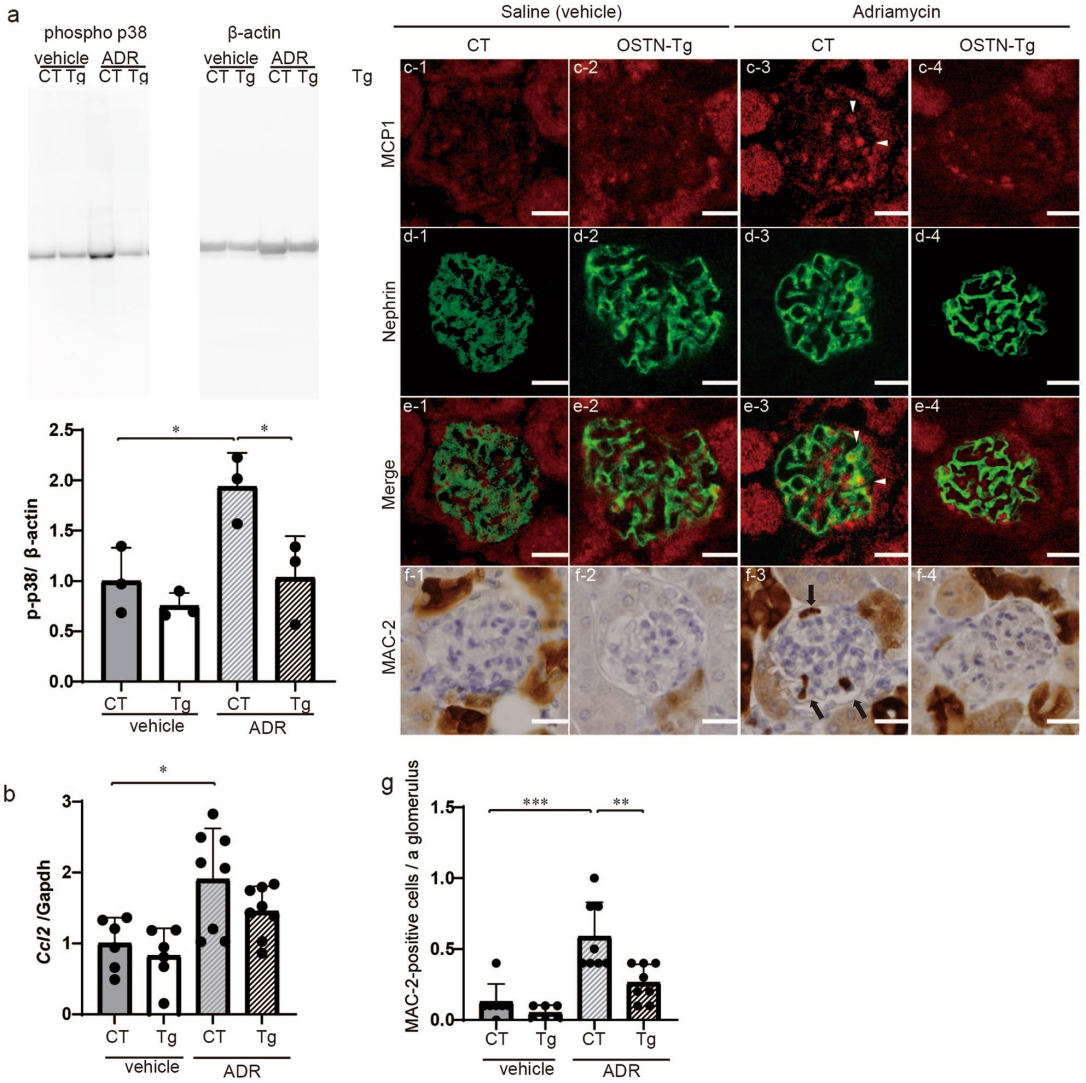
ADR-induced inflammation was ameliorated in OSTN-Tg mice. (**a**) Glomerular p38 MAPK phosphorylation in four groups at 10 weeks of age. The grouping of gels cropped from the same lines of the same gel. Full-length blots/gels are presented in Supplementary Fig. S13a. (**b**) Glomerular mRNA expression levels of *Ccl2* in four groups at 10 weeks of age. (**c**–**e**) Immunofluorescent studies for MCP1 (**c**; red), nephrin (d; green), and merged images (**e**) in four groups. (**f**,**g**) Immunohistochemical findings of MAC-2 (**f**), a macrophage marker, and the number of MAC-2-positive cells in glomeruli in four groups (**g**). phospho p38, phosphorylated p38 MAPK; CT, control mice; Tg, OSTN-Tg mice. Data are mean ± SD. *P < 0.05, **P < 0.01, ***P < 0.001 by one-way ANOVA analysis.

We examined glomerular phosphorylated p38 MAPK by Western blotting, glomerular *Ccl2* mRNA expression, and MCP1 and MAC-2 by immunostaining to assess inflammation in ADR nephropathy (Fig. 3). Phosphorylated p38 MAPK in ADR CT mice was significantly upregulated compared to that in vehicle-treated mice, and this increase was significantly attenuated in ADR Tg mice (Fig. 3a, Fig. S13a). Glomerular *Ccl2* mRNA was increased in ADR CT mice compared to that in vehicle-treated CT mice, and this upregulation was tended to reduce in ADR Tg mice (Fig. 3b). No difference was observed in the expression of other genes in glomeruli (Fig. S2). Immunofluorescent staining showed that MCP1 was upregulated in ADR CT mice, and MCP1 and nephrin staining were co-stained in all four groups, indicating that the expression site of MCP1 in glomeruli was podocytes (Fig. 3c–e). In addition, the number of MAC-2-positive cell, presumably macrophage, infiltrating into glomeruli of ADR CT mice was higher than that of the other three groups (Fig. 3f,g). These findings indicated that ADR induced phosphorylation of p38 MAPK, upregulation of *Ccl2* and infiltration of MAC-2-positive cells, all of which were ameliorated by circulating OSTN.

### Systemic deletion of OSTN aggravates adriamycin nephropathy

**Figure 4:**
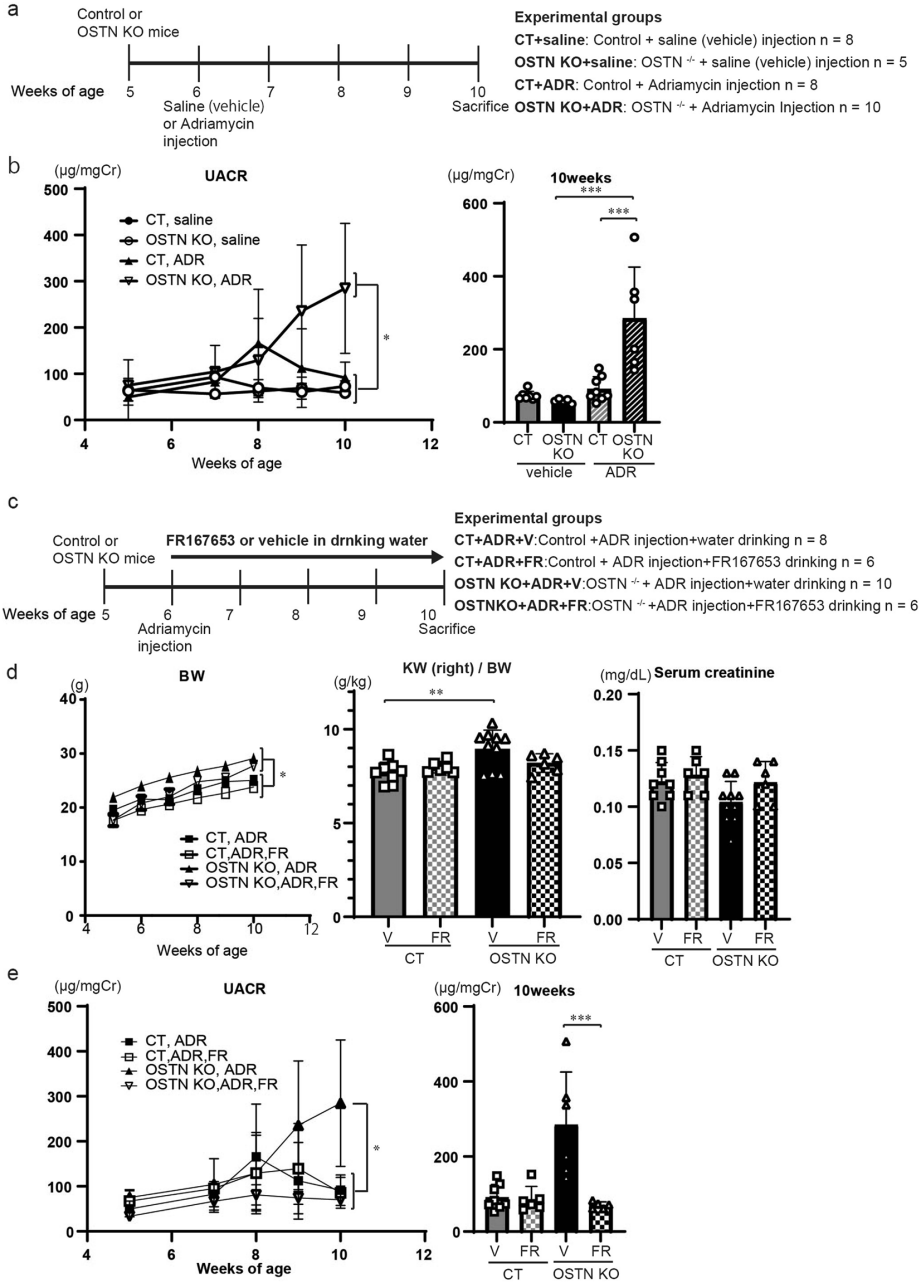
FR167653, a p38 MAPK inhibitor, reduced ADR-induced albuminuria in OSTN KO mice. (**a**) Schematic of the experimental protocol. OSTN KO or control mice were injected intravenously with saline or ADR at 6 weeks of age. CT saline and CT ADR mice were used as reference groups shown in Fig. 1. (**b**) Urinary albumin creatinine ratio at weeks 5, 7, 8, 9 and 10 (n = 5–10 per group). (**c**) Schematic of the experimental protocol. OSTN KO or control mice were treated with FR167653 or vehicle and injected intravenously with ADR at 6 weeks of age. CT ADR and OSTN KO ADR mice were used as reference groups shown in Fig. 1 and Fig. 4a, respectively. (**d**) The BW at weeks 5, 6, 7, 8, 9 and 10 and right kidney weight, serum albumin, serum creatinine levels at 10 weeks of age (n = 6–10 per group). *P < 0.05, CT + V vs. OSTN KO + V, or CT + FR vs. OSTN KO + FR. **P < 0.01 by one-way ANOVA analysis. (**e**) Urinary albumin creatinine ratio at weeks 5, 7, 8, 9 and 10 (n = 6–10 per group). UACR: OSTN KO 284.8 ± 140.5 μg/mgCr vs. OSTN KO-FR 69.8 ± 8.2 μg/mgCr. Data are mean ± SD. *P < 0.05, **P < 0.01 and ***P < 0.001 by one-way ANOVA analysis. *BW* body weight, *V* vehicle, *FR* FR167653, *CT* control mice, *OSTN KO* OSTN KO mice, *UACR* urinary albumin creatinine ratio.

Systemic OSTN KO mice were similarly injected with ADR to assess the role of circulating OSTN (Fig. 4, Fig. S3), and the BW of OSTN KO mice was significantly higher than that of CT mice regardless of ADR or vehicle injection (Fig. S3a). There were no significant differences in creatinine levels, but the kidney weight of OSTN KO mice was significantly heavier than that of CT mice (Fig. S3a). Systolic blood pressure in ADR OSTN KO mice was significantly higher than that in ADR CT mice (Fig. S3b). UACR at 10 weeks of age of ADR-injected OSTN KO (ADR OSTN KO) mice were significantly higher than those of ADR CT mice (Fig. 4b). These biological findings indicated that the deletion of circulating OSTN exacerbates ADR-induced albuminuria.

We examined microscopic findings of CT and OSTN KO mice (Fig. S4). There were no differences in glomerular PAS stain between CT and OSTN KO mice regardless of ADR (Fig. S4a). Desmin staining area was increased in ADR CT mice and tended to increase further in ADR OSTN KO mice (Fig. S4b,e). Foot process effacements and width of foot processes in ADR OSTN KO mice were similar to those in ADR CT mice in electron microscopic findings (Fig. S4c,d,f). Glomerular p38 MAPK phosphorylation in ADR OSTN KO mice was significantly upregulated compared to that in ADR CT mice, and *Ccl2* mRNA expression and MCP1 staining in ADR OSTN KO mice tended to increase, but there was no significant difference between ADR CT and ADR OSTN KO mice (Fig. S5a,b, Fig. S13d). Other glomerular gene expressions were not significant different between ADR CT and ADR OSTN KO mice except for *Acta2* and *Ostn* (Fig. S6). Immunofluorescent staining illustrated that nephrin was reduced in ADR OSTN KO mice, and that MCP1 and nephrin staining were merged in these mice (Fig. S5c–e). MAC-2-positive cell infiltration was similar between ADR CT and ADR OSTN KO mice (Fig. S5f,g). These findings indicated that ADR-induced podocyte injury was observed in OSTN KO mice to the same extent as in CT mice. We speculate that plasma concentration of OSTN in CT mice is so low that OSTN KO mice exhibit mild changes compared to CT mice.

### Inhibition of p38 MAPK mitigates adriamycin nephropathy

Next, to confirm whether p38 MAPK is an essential factor for ADR-induced podocyte injury, we treated CT and OSTN KO mice with FR167653, a p38 MAPK inhibitor, to identify whether p38 MAPK was an essential factor in ADR-induced podocyte injury (Fig. 4). Figure 4c shows the protocol of ADR CT and OSTN KO mice treated with FR167653. The BW of ADR OSTN KO mice was significantly increased compared to that in ADR CT mice regardless of FR treatment (Fig. 4d). There were no significant differences in serum creatinine levels, but the kidney weight of OSTN KO mice was significantly increased compared to that of CT mice (Fig. 4d). ADR OSTN KO vehicle mice showed elevated systolic blood pressure compared to ADR CT vehicle mice, while FR treatment did not change blood pressure in ADR OSTN KO mice (Fig. S7). FR167653 significantly reduced UACR in ADR OSTN KO mice at 10 weeks of age (Fig. 4e).

We examined PAS staining, immunohistochemical study for desmin and electron microscopic data to assess podocyte injury morphologically (Fig. S8). There were no differences in glomerular PAS staining between both CT and OSTN KO mice regardless of FR167653 (Fig. S8a). The positive area of desmin was significantly reduced in the FR treatment groups (Fig. S8b,e). Foot process effacements observed in both ADR CT and ADR OSTN KO mice were ameliorated in the FR167653 treatment groups (Fig. S8c,d). The increases in thickening of GBM and width of foot process by ADR treatment were significantly mitigated in FR167653-treated groups in both CT and OSTN KO mice (Fig. S8f). These histological findings indicated that p38 MAPK inhibition ameliorates ADR-induced podocyte injury.

We examined glomerular p38 MAPK phosphorylation, glomerular mRNA expression of *Ccl2*, and immunostaining of MCP1 and MAC-2 (Fig. S9). Western blotting showed that FR167653 treatment suppressed glomerular p38 MAPK phosphorylation in both CT and OSTN KO mice (Fig. S9a, Fig. S13e). FR167653 significantly suppressed ADR-induced glomerular *Ccl2* mRNA expression (Fig. S9b) and immunofluorescent staining of MCP1 in both CT and OSTN KO mice (Fig. S9c). The glomerular mRNA expression of *Col1a1*, *Ctgf*, *Npr1* and *Npr3* in ADR OSTN KO vehicle mice was significantly decreased by the treatment of FR167653 (Fig. S10). Nephrin was recovered in FR167653-treated OSTN KO mice, and MCP1 and nephrin staining colocalized in all four groups (Fig. S9c–e). FR167653 significantly ameliorated the infiltration of MAC-2-positive cells in glomeruli of CT and OSTN KO mice (Fig. S9f,g). These results suggest that FR167653 inactivates p38 MAPK in glomeruli and consequently suppresses *Ccl2* expression and MAC-2-positive cell infiltration in both ADR CT and ADR OSTN KO mice.

### The effects of OSTN is mainly dependent on NPR-C

**Figure 5:**
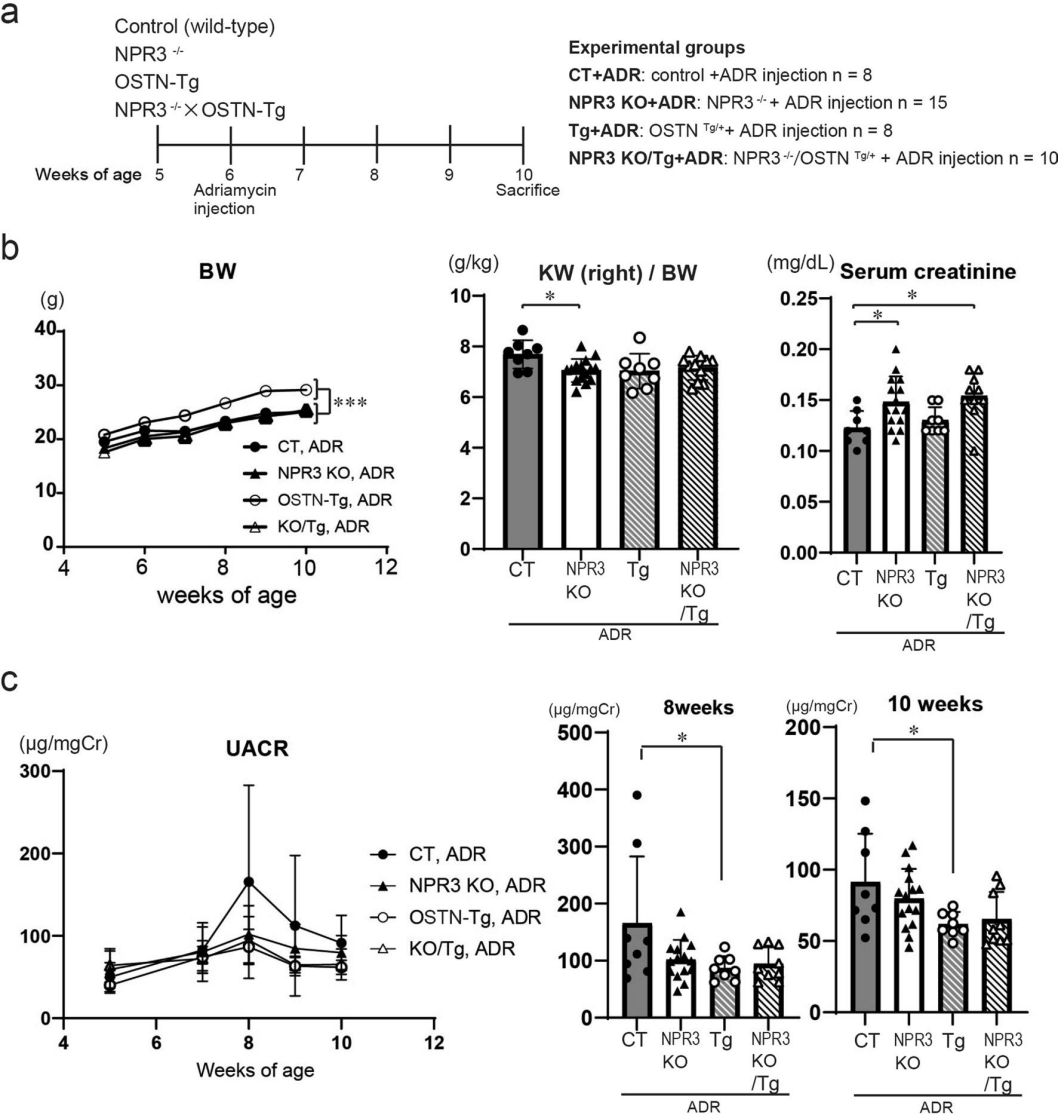
Urinary albumin levels were lower in NPR3 KO mice than in control mice under ADR nephropathy. (**a**) Schematic of the experimental protocol. OSTN-Tg, NPR3 KO, NPR3 KO/OSTN-Tg, or control mice were injected intravenously with saline or ADR at 6 weeks of age. CT ADR and Tg ADR mice were used as reference groups shown in Fig. 1. (**b**) The BW at weeks 5, 6, 7, 8, 9 and 10 and serum creatinine levels at 10 weeks of age (n = 8–15 per group). ***P < 0.001, OSTN-Tg vs. NPR3 KO, NPR3 KO/OSTN-Tg or control by one-way ANOVA analysis. (**c**) Urinary albumin creatinine ratio at weeks 5, 7, 8, 9, 10 (n = 8–15 per group); CT, control mice; Tg, OSTN-Tg mice. Data are mean ± SD. *P < 0.05, by one-way ANOVA analysis.

OSTN has been reported to be a selective ligand for the clearance receptor, NPR-C, and to consequently enhance NP signaling. To investigate whether podocyte protective effects of circulating OSTN are dependent on NPR-C, we used NPR3 KO mice (*Npr3*^−/−^/*Ostn*^+/+^ mice), double mutant mice carrying the *Ostn*-transgene depleted of the *Npr3* gene (*Npr3*^−/−^/*Ostn*^Tg/+^ mice), and OSTN-Tg and CT mice, and all four groups were treated with ADR (Fig. 5a). The BW of OSTN-Tg mice was higher than that of CT mice, but the gain in BW was decreased in *Npr3*^−/−^/*Ostn*^Tg/+^ mice (Fig. 5b). Systolic blood pressure was not different among the four groups (Fig. S11). Serum creatinine was significantly higher in NPR3 KO mice than in control, and this increase was not altered in *Npr3*^−/−^/*Ostn*^Tg/+^ mice (Fig. 5b). UACR in ADR CT mice peaked at 8 weeks of age, whereas UACR decreased in Tg mice, and tended to decrease in both NPR3 KO mice and *Npr3*^−/−^/*Ostn*^Tg/+^ mice (Fig. 5c).

**Figure 6:**
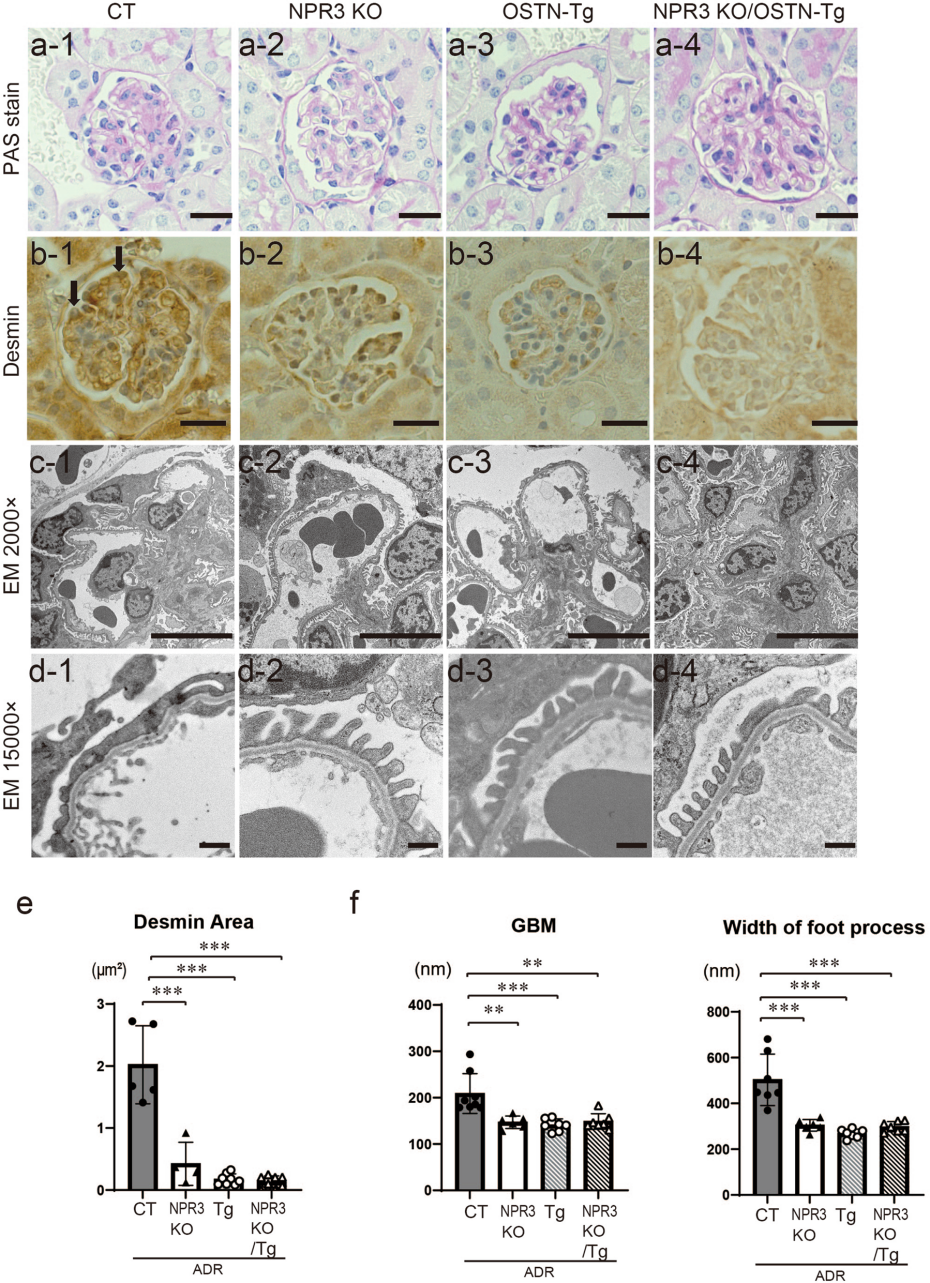
ADR-induced NPR3 KO mice exhibited podocyte protective effects similar to OSTN-Tg mice. (**a**) Light microscopic analysis stained with PAS. Bars, 20 μm. (**b**) Immunohistochemical study for desmin (magnification, × 400). Bars, 20 μm. (**c**,**d**) Electron microscopic analysis with lower magnification (**c**; magnification, × 2000, Bars, 10 μm), and higher magnification (**d**; magnification, × 15,000, Bars, 500 nm). (**e**) Desmin-positive areas were analyzed. (**f**) Thickness of glomerular basement membrane (GBM) and width of foot process effacements were measured. GBM thickness: *PAS* periodic acid-Schiff, *EM* electron microscopy, *CT* control mice, *Tg* OSTN-Tg mice. Data are mean ± SD. *P < 0.05, **P < 0.01, ***P < 0.001 by one-way ANOVA analysis.

We examined PAS staining, immunohistochemical study for desmin and electron microscopic data to assess podocyte injury morphologically (Fig. 6). PAS stain showed no differences in glomerular findings in 4 groups (Fig. 6a). The positive area of desmin in ADR CT was significantly reduced in other 3 groups (Fig. 6b,e). Foot process effacements, thickening of GBM and width of foot process observed in ADR CT were ameliorated in other 3 groups (Fig. 6c,d,f). These histological findings indicated that OSTN ameliorates ADR-induced podocyte injury via NPR3.

**Figure 7:**
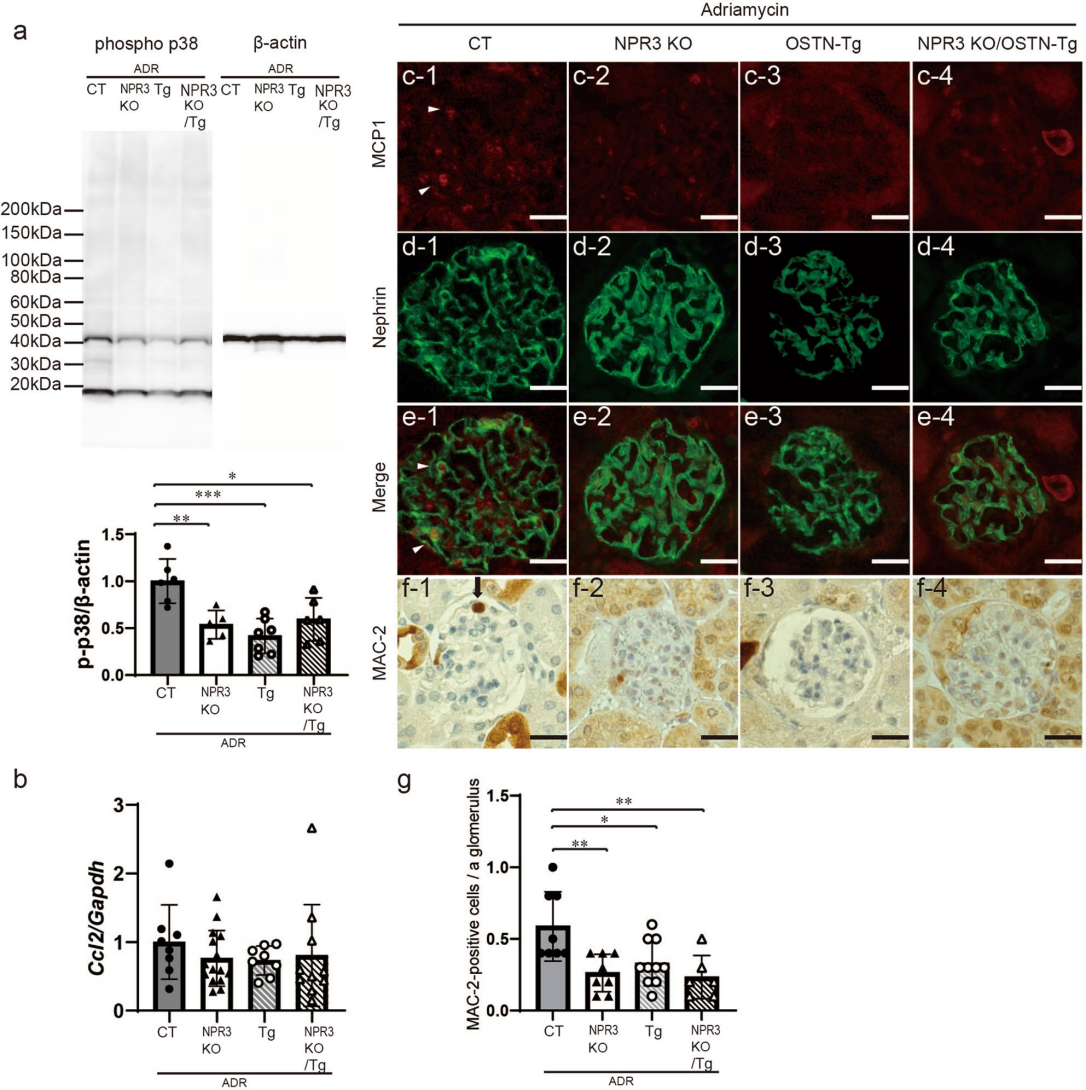
p38 MAPK phosphorylation in NPR3 KO mice was lower than that of control mice. (**a**) Glomerular p38 MAPK phosphorylation in four groups at 10 weeks of age. The grouping of gels cropped from the same lines of the same gel. Full-length blots/gels are presented in Supplementary Fig. S13b. (**b**) Glomerular mRNA expression levels of *Ccl2* in four groups at 10 weeks of age. (**c**–**e**) Immunofluorescent studies for MCP1 (**c**; red), nephrin (**d**; green), and merged images (**e**) in four groups. (**f**,**g**) Immunohistochemical findings of MAC-2 (**f**), a macrophage marker, and the number of MAC-2-positive cells in glomeruli in four groups (**g**). Phospho p38, phosphorylated p38 MAPK; CT, control mice; Tg, OSTN-Tg mice. Data are mean ± SD. *P < 0.05, **P < 0.01, ***P < 0.001 by one-way ANOVA analysis.

We examined glomerular p38 MAPK phosphorylation, glomerular mRNA expression of *Ccl2*, and immunostaining of MCP1 and MAC-2 (Fig. 7). Western blotting showed that phosphorylated p38 MAPK in ADR CT mice was significantly upregulated compared to that in other 3 groups (Fig. 7a, Fig. S13b). There were no differences of glomerular *Ccl2* mRNA expression and immunofluorescent staining of MCP1 in 4 groups (Fig. 7b–e) but MAC2-positive cells per a glomerulus of ADR CT were increased compared to those of ADR NPR3-KO, ADR Tg and ADR double mutant (Fig. 7f,g). In NPR3 KO mice, the expression of *Col1a1*, *Fn, Npr1* and *Npr2* was decreased in glomeruli, regardless of whether or not they carried the OSTN-transgene (Fig. S12). These results showed that deletion of NPR3 reduced ADR-induced podocyte injury to the same extent as that in OSTN-Tg mice. Additionally, we showed that *Npr3*^−/−^/*Ostn*^Tg/+^ mice exhibited phenotypes similar to those of Tg and NPR3 KO mice without additive improvement, indicating that the effects of OSTN are mainly dependent on NPR3, and enhancement of NP signaling by OSTN suppresses p38 MAPK activation in podocytes.

### Activation of GC-A signaling by OSTN suppresses p38 MAPK, *Ccl2* and *Des* in podocytes

**Figure 8:**
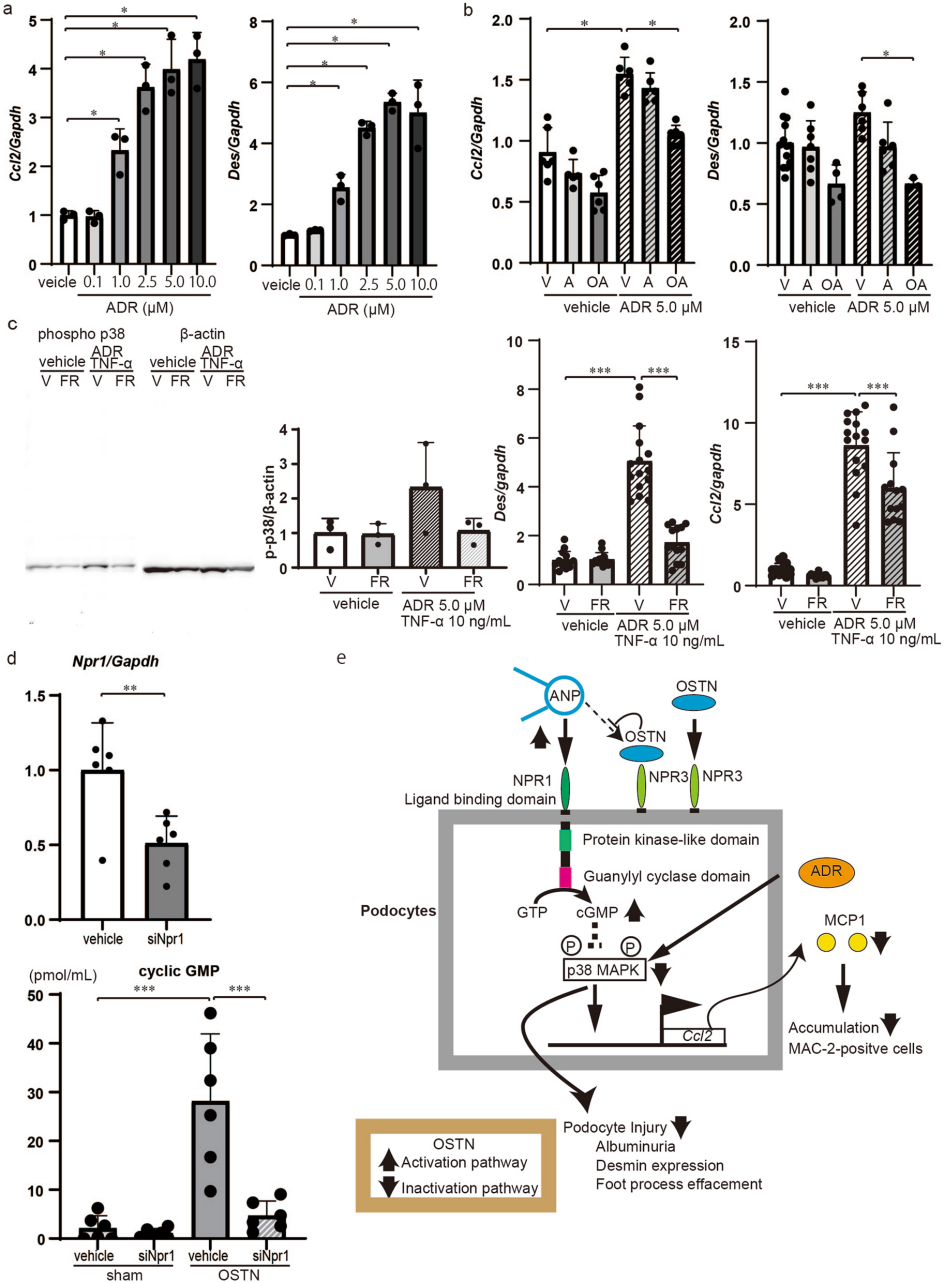
OSTN increased intracellular cGMP via GC-A/NPR1 and consequently decreased phosphorylation of p38MAPK. (**a**) MPC5 (mouse podocyte cells) were stimulated by ADR at various concentrations to assess mRNA expression of *Ccl2* and *Des.* (**b**) ADR-stimulated MPC5 were treated by OSTN and ANP to evaluate mRNA expression of *Ccl2* and *Des.* (**c**) Phosphorylation of p38 MAPK at 2 h and mRNA expression of *Des* and *Ccl2* at 24 h in MPC5 stimulated with ADR and TNF-α and simultaneously with FR167653 or vehicle. The grouping of gels cropped from the same lines of the same gel. Full-length blots/gels are presented in Supplementary Fig. S13c. (**d**) Expression of *Npr1* mRNA in MPC5 transfected with Npr1 siRNAs or control siRNAs. Intracellular cGMP in MPC5 transfected with Npr1 siRNAs or control siRNAs and subsequently treated with OSTN or vehicle. (**e**) Proposed mechanisms of OSTN-protective roles in ADR-stimulated podocytes. NPR-C has no intrinsic enzymatic activity and decreases local concentrations of natriuretic peptides through receptor-mediated homeostatic internalization and degradation. OSTN binds to NPR-C with high affinity and enhances guanylyl cyclase A (GC-A)/NPR1 signaling in podocytes by inhibiting natriuretic peptide degradation. Activation of GC-A leads to the synthesis of cGMP, and the physiological effects of natriuretic peptides are mediated by three cGMP-binding proteins: cGMP-dependent protein kinase, cGMP-regulated phosphodiesterase, and cyclic nucleotide-dependent ion channel. The physiological effects of natriuretic peptides in podocytes inhibit phosphorylation of p38 MAPK, which was an important mediator of ADR-induced podocyte injury, and downregulate Ccl2 mRNA. As a result, it ameliorates podocyte injury such as albuminuria, upregulation of desmin, and loss of foot projections. V, vehicle; A, 10^–6^ M ANP; OA, 10^–6^ M OSTN and 10^–6^ M ANP; FR, FR167653. Data are mean ± SD. *P < 0.05, **P < 0.01, ***P < 0.001 by one-way ANOVA analysis.

To determine the role of ADR in podocytes in vitro, we stimulated mouse podocytes (MPC5) with ADR. Expression of *Ccl2* and *Des* mRNA was upregulated by ADR stimulation in MPC5 dose-dependently (Fig. 8a). Stimulation with 10^–6^ M ANP induced a downward trend of *Ccl2* and *Des* mRNA expression in ADR-treated MPC5, but it was not significant; addition of 10^–6^ M OSTN to 10^–6^ M ANP significantly suppressed both *Ccl2* and *Des* mRNA expression in MPC5 (Fig. 8b), indicating that OSTN has additive effects on inhibition of *Ccl2* and *Des* mRNA expression.

Next, to investigate the significance of p38 MAPK inhibition and the mechanism of action of OSTN in podocytes, we analyzed the expression of mRNA expression, the intracellular cGMP through GC-A (*Npr1*), and the effects of OSTN on ADR-stimulated MPC5. Western blotting showed that ADR and TNFα stimulation upregulated p38 MAPK, and FR1657653 treatment suppressed this increase. ADR and TNFα stimulation also increased *Des* and *Ccl2* mRNA expression in MPC5, which was suppressed by FR167653 (Fig. 8c, Fig. S13c). OSTN increased intracellular cGMP in MPC5, and this increase was abolished in MPC5 in which GC-A was knocked down by siRNA (Fig. 8d). Figure 8e illustrates our proposed mechanism; OSTN binds to NPR-C, a NP clearance receptor, and enhances GC-A signaling pathway in podocytes. Activation of the GC-A signaling pathway by OSTN suppresses p38 MAPK phosphorylation, which was an important molecule for ADR-induced podocytes, downregulates *Ccl2* mRNA, and ameliorates podocyte injury (Fig. 8e).

## Discussion

In the present study, we investigated the biological and histological findings, gene expression and signaling pathway including p-38 MAPK among ADR-induced CT, OSTN-Tg, OSTN KO and *Npr3*^−/−^/*Ostn*^Tg/+^ mice in vivo. In addition, we revealed that OSTN has inhibitory effects on p38 MAPK phosphorylation and *Ccl2* expression in vitro, indicating that OSTN enhances the GC-A signaling pathway and suppresses ADR-stimulated p38 MAPK activation. We presented that OSTN has reno-protective effects on ADR nephropathy.

ADR-induced OSTN-Tg mice reduced UACR, and in contrast, ADR-induced OSTN KO mice increased UACR. These results indicated that circulating OSTN ameliorates ADR-induced albuminuria. In addition, UACR was decreased in both ADR-induced NPR3 KO and *Npr3*^−/−^/*Ostn*^Tg/+^ mice as well as Tg mice. These results may be due to the circumstance that OSTN is inactivated in NPR3, a clearance receptor. Electron microscopy illustrated that the phenotypes of ADR nephropathy are affected by circulating OSTN. The area of desmin, a podocyte injury marker^5^, in OSTN KO mice was larger than that in CT and Tg mice. In vitro, the expression of desmin was upregulated in ADR-stimulated podocytes, which was suppressed by OSTN and ANP treatments, indicating that OSTN has a therapeutic effect on ADR-induced podocyte injury.

ADR activates various molecular signals. MCP1 (*Ccl2*), a cytokine involved in macrophage chemotaxis, has been reported to be upregulated in ADR nephropathy by albumin excess in renal tubules^2^, NFκ-B activation^31,36^, and mononuclear cell infiltration in the kidney^35^. Recent reports have revealed that MCP1 is increased in focal segmental glomerulosclerosis in humans and murine podocytes, and that TNF-α induces MCP1 expression, which is an important mediator of ADR-induced nephropathy^34^. We focused on glomerular mRNA expression of *Ccl2*, MCP1 localization and macrophage infiltration in order to assess inflammatory changes in ADR nephropathy. Glomerular mRNA expression of *Ccl2* was suppressed in Tg mice and conversely upregulated in OSTN KO mice. Fluorescent immunostaining showed MCP1 and nephrin were colocalized, indicating that MCP1 is mainly expressed in podocytes of ADR nephropathy. The intensity of MCP1 immunostaining was strong in OSTN KO mice, but weak in Tg mice. In vitro, *Ccl2* is upregulated in ADR-stimulated podocytes, which is inhibited by the treatment with ANP and OSTN. These results indicate that circulating OSTN suppresses ADR-induced MCP1 upregulation in podocytes. Macrophage infiltration is one of the phenotypes of ADR-induced nephropathy^3,21^ and predictor of subsequent disease progression^4^. Macrophage infiltration of glomeruli in OSTN KO mice was more prominent than that in CT and Tg mice. This is consistent with the results of MCP1 (*Ccl2*) expression and immunostaining.

MCP1 is regulated by p38 MAPK in podocytes^30^, ADR nephropathy^35^, murine anti-glomerular basement membrane nephritis^31^, and acute folate nephropathy^33^. Inhibition of p38 MAPK ameliorates ADR nephropathy^29,37^. In vitro, p38 MAPK was activated in MPC5 stimulated with TNF-α and ADR, and FR167653 suppressed its activation. FR167653 suppressed mRNA expression of *Ccl2* and *Des*. Next, we investigated whether the podocyte-protective role of circulating OSTN was dependent on p38 MAPK activation. OSTN-Tg mice downregulated p38 MAPK phosphorylation in glomeruli, whereas OSTN KO mice upregulated p38 MAPK phosphorylation. FR167653-treated CT and OSTN KO mice improved ADR nephropathy phenotypes. These findings suggest that circulating OSTN inhibited p38 MAPK activation and consequently ameliorated ADR nephropathy.

OSTN binds with high affinity to NPR-C^18^, the clearance receptor of NPs, and inhibits degradation of NPs, thereby increased circulating NPs enhancing GC-A signaling pathway in endothelial cells^20^ and myoblasts^21^. OSTN is reported to induce skeletal overgrowth thorough NPR-B/CNP signaling in OSTN-Tg mice^24^. This report is consistent with our findings of the BW in OSTN-Tg mice is higher than CT mice, but the finding that the BW of OSTN-KO mice is higher than that of control mice remains unclear. The roles of OSTN in renal cells including podocytes are not reported yet. We revealed that OSTN induced intracellular cGMP elevation in podocytes via GC-A. In vivo, we demonstrated that NPR3 KO mice ameliorated ADR nephropathy. These results are consistent with the proposed mechanism of protective effects of OSTN on ADR nephropathy. Although ANP activates p38 MAPK phosphorylation in adipocytes^38^, we previously reported that GC-A signaling pathway suppresses the activation of p38 MAPK^14^, and the suppression of p38 MAPK in this ADR nephropathy reduces mRNA expression of *Ccl2* and ameliorates podocyte injury (Fig. 8e). The difference in p38 MAPK activation by NPs may be due to cell types.

In conclusion, we elucidated that the circulating OSTN suppressed p38 MAPK activation in podocytes, and that OSTN enhances GC-A signaling, thereby ameliorating ADR nephropathy. Administration of OSTN can be a potential therapeutic option for albuminuria.

## Methods

### Reagents and antibodies

Adriamycin was obtained from Sigma Aldrich (St. Luis, MO). FR167653, p38α MAPK inhibitor, was kindly provided by Astellas Pharma Inc. (Tokyo, Japan). Primary antibodies used for immunohistochemical studies and Western blotting were mouse anti-NPR-C (1:100, sc-515449; SantaCruz, Dallas, TX), goat anti-nephrin (1:100, AF3159; R&D Systems, Minneapolis, MN), rabbit anti-monocyte chemotactic protein-1 (1:100, ab25124; Abcam, Cambridge, UK), rabbit anti-phospho-p38 MAPK (1:1000, #9211; Cell Signaling Technology, Boston, MA), mouse anti-desmin (1:150, M0760; DAKO, Tokyo, Japan), mouse anti-β actin (1:1000, A5411; Sigma-Aldrich), and rat anti-MAC-2 (1:100, CL8942F; Cedarlane, CA).

### Experimental animals and treatments

All animal experiments were performed in accordance with the Fundamental Guidelines for Proper Conduct of Animal Experiment and Related Activities in Academic Research Institution, and were approved by the Animal Experimentation Committee of Kyoto University Graduate School of Medicine (Approval number; MedKyo 20186) and according to the ARRIVE guidelines^39^. OSTN-transgenic (Tg) mice, which express *Ostn* specifically in the liver under control of the human serum amyloid-P (SAP) component promoter have been reported previously^24^. Systemic *Ostn* KO^25^ and *Npr3*-knockout (NPR3 KO) mice^11^ have been reported previously. These mice were backcrossed with BALB/c mice three times, as they were generated on a C57BL/6J background. Control mice (+/+) were also backcrossed with BALB/c mice three times from C57BL/6J background. We prepared double mutant mice (*Npr3*^−/−^/*Ostn*^Tg/+^ mice) by crossing NPR3 KO mice and Tg mice. Male mice were used for all experiments.

Saline (vehicle) or ADR was administered to 6-week-old mice at a dose of 8 mg/kg body weight (BW) via tail-vein injection. Mice were sacrificed at 4 weeks after ADR injection. FR167653 was administered at a concentration of 0.66 mg/mL in drinking water^14^. Saline- or ADR-administered control mice and OSTN KO mice were used as reference groups for all experiments. Urine samples were collected from metabolic cages (Shinano Manufacturing, Tokyo, Japan) for 24 h at 5, 7, 8, 9, and 10 weeks of age, and urine volumes were measured. Blood and kidney samples were harvested at 10 weeks of age. Urinary albumin levels were measured using the albumin ELISA kit (FUJIFILM Wako Shibayagi Corporation, Shibukawa, Japan). Serum creatinine and urinary creatinine were measured by enzymatic methods (SRL, Tokyo, Japan).

### Renal histology and electron microscopic analysis

Histological and electron microscopic examinations were performed as described previously^40^. Periodic acid-Schiff (PAS) stained kidney samples were examined by light microscopy. Desmin-positive areas were measured using MetaMorph Software (Molecular Devices, Sunnyvale, CA)^40^. Electron microscopic examination was performed using an electron microscope (H-7600, Hitachi, Tokyo, Japan)^40^. We averaged the number of MAC-2-positive cells per 10 glomeruli in each subject. We used Photoshop ver. 22.5.1 and Illustrator ver. 25.4.1 as image processing software packages.

### Immunofluorescent and immunohistochemical studies

Immunofluorescent studies for nephrin, NPR-C and MCP1 were performed as described previously^13^. Briefly, cryostat sections were incubated with goat anti-nephrin antibody or rabbit anti-MCP1 antibody, and then incubated with fluorescein isothiocyanate (FITC)-labeled secondary antibody. Immunohistochemical analysis for NPR-C, desmin, and MAC-2 were as previously described with some modifications^13^.

### Cell experiments

MPC-5 cells, an immortalized mouse podocyte cell line^41,42^, were used for in vitro experiments. Briefly, MPC-5 cells were cultured with RPMI 1640 medium (Sigma-Aldrich) supplemented with 10% fetal bovine serum (FBS; Biowest, Nuaille, France) and interferon-gamma (IFN-γ; PeproTech, Cranbury, NJ) before differentiation. MPC-5 cells were differentiated with incubation in IFN-γ (–) media at 37 °C for 2 weeks before stimulation. Differentiated podocytes were stimulated with ADR for 24 h and treated with 10^–6^ M ANP (Peptide institute. Inc., Osaka, Japan) and/or 10^–6^ M OSTN (Phoenix Pharmaceuticals, Inc. Burlingame, CA) and then were harvested for RNA analysis. To assess the roles of p38 MAPK in ADR-stimulated MPC5, differentiated cells were stimulated with 5.0 μM ADR and 10 ng/mL TNF-α (R&D Systems, Minneapolis, MN) simultaneously with 10 μM FR167653 or vehicle for 2 or 24 h, and were then harvested for Western blotting or RNA analysis. Differentiated podocytes were transfected with Npr1, or control siRNA using the Nucleofector Kit for MPC5 (Lonza, Basel, Switzerland) as described previously^43^. Transfected cells were incubated with 50 mM 3-isobutyl-1-methylxanthine (IBMX; Nacalai Tesque, Kyoto, Japan) for 20 min and were then treated with 10^–6^ M OSTN or vehicle for 10 min to measure intracellular cGMP concentration using the Cayman ELISA Kit (Ann Arbor, MI).

### Glomerular RNA, protein extraction and real-time RT-PCR

Glomeruli were isolated by graded sieving methods as described previously^13^. RNA and protein extraction were performed using AllPrep DNA/RNA/protein kits (QIAGEN, Hilden, Germany). Quantitative real-time PCR was performed using the StepOnePlus System (Thermo Fischer Scientific, Waltham, MA), as descried previously^40^. Expression levels of *Ccl2*, *Des*, and *Npr1* mRNAs were evaluated. Primer and probe sets were described previously^13^, and in Table S1.

### Western blotting

Western blotting was performed as previously described^13^. Filters on isolated cell extracts were incubated with rabbit anti-phospho-p38MAPK and mouse anti-β actin antibodies. Immunoblots were developed using horseradish peroxidase-linked donkey anti-rabbit or anti-mouse antibodies and a chemiluminescent kit.

### Statistical analysis

Values are expressed as the mean ± standard deviation (SD) and were analyzed with Graph Prism software (Version 9.00, GraphPad, San Diego, CA). Unpaired Student’s *t* test was used to compare differences between the two groups, whereas comparisons of more than two groups were performed by one-way ANOVA with a Tukey post hoc test. Statistical significance was set at *P* < 0.05.

## Supplementary Information

Supplementary Information.

## Data Availability

The datasets used and/or analyzed during the current study are available from the corresponding author on reasonable request.
