# Supplementary material for: Osteocrin ameliorates adriamycin nephropathy via p38 mitogen-activated protein kinase inhibition
Source: Sci Rep. 2021 Nov 8;11:21835. doi: 10.1038/s41598-021-01095-8 (PMC8575949; doi:10.1038/s41598-021-01095-8)
Supplement: Supplementary file 1 — Supplementary Information. [file 41598_2021_1095_MOESM1_ESM.pdf]

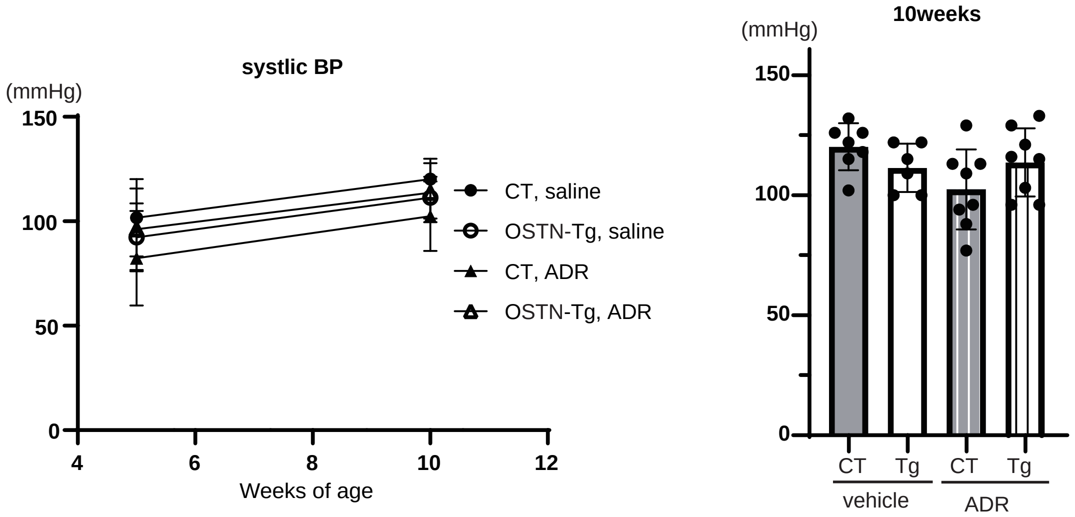

Supplementary Figure S1 Systolic blood pressure at weeks 5 and 10 (n = 7-8 per group).

CT + saline vs. CT + ADR by one-way ANOVA analysis.

BP, blood pressure; CT, Control mice; Tg, OSTN-Tg mice. Data are mean ± SD.

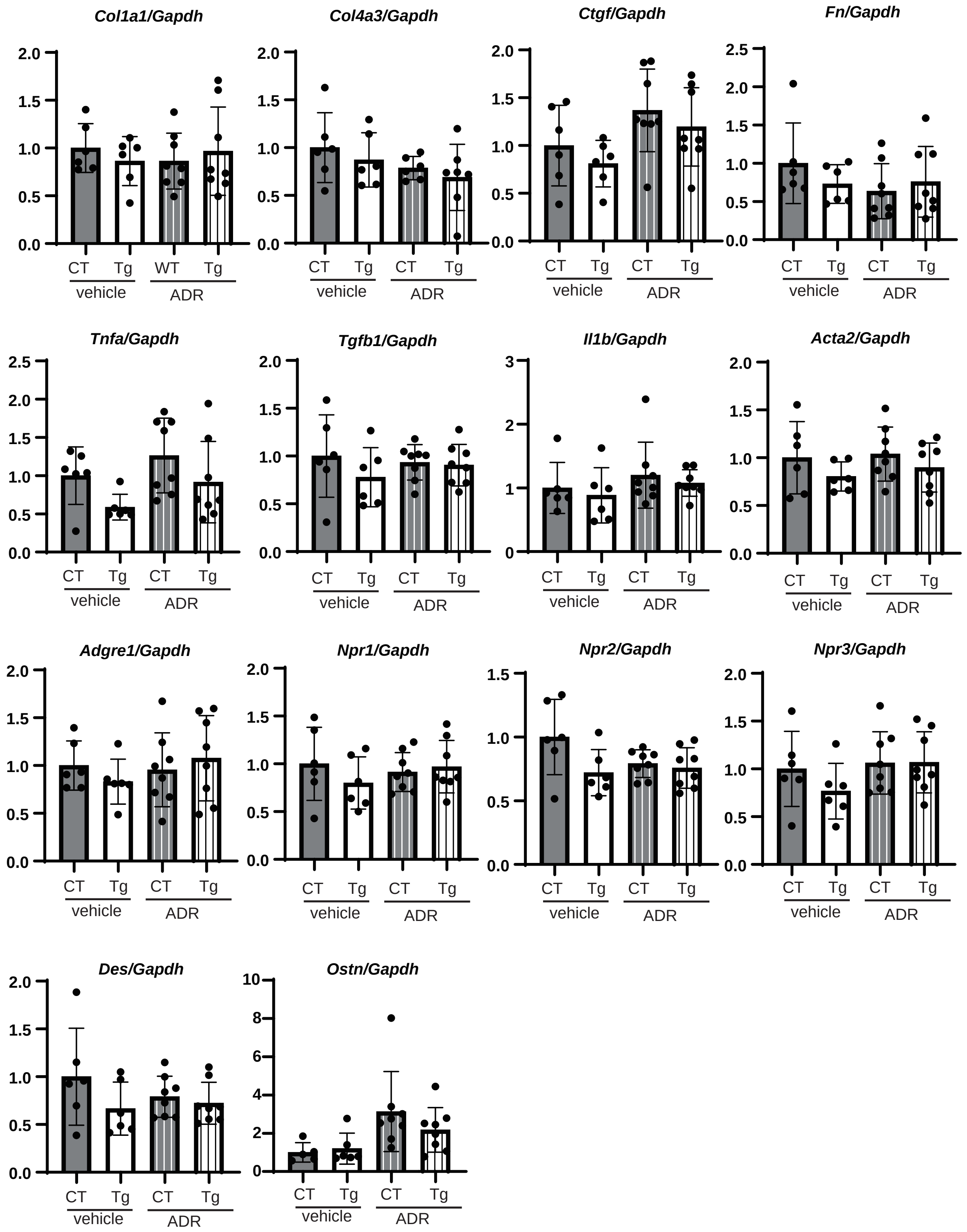

Supplementary Figure S2 The glomerular expression of *Col1a1* , *Col4a3* , *Ctgf* , *Fn* , *Tnfa* , *Tgfb1* , *Il1b* , *Acta2* , *Adgre1* , *Npr1* , *Npr2* , *Npr3 and Des, Ostn* in OSTN-Tg mice and control mice under ADR nephropathy. Data are mean ± SD.

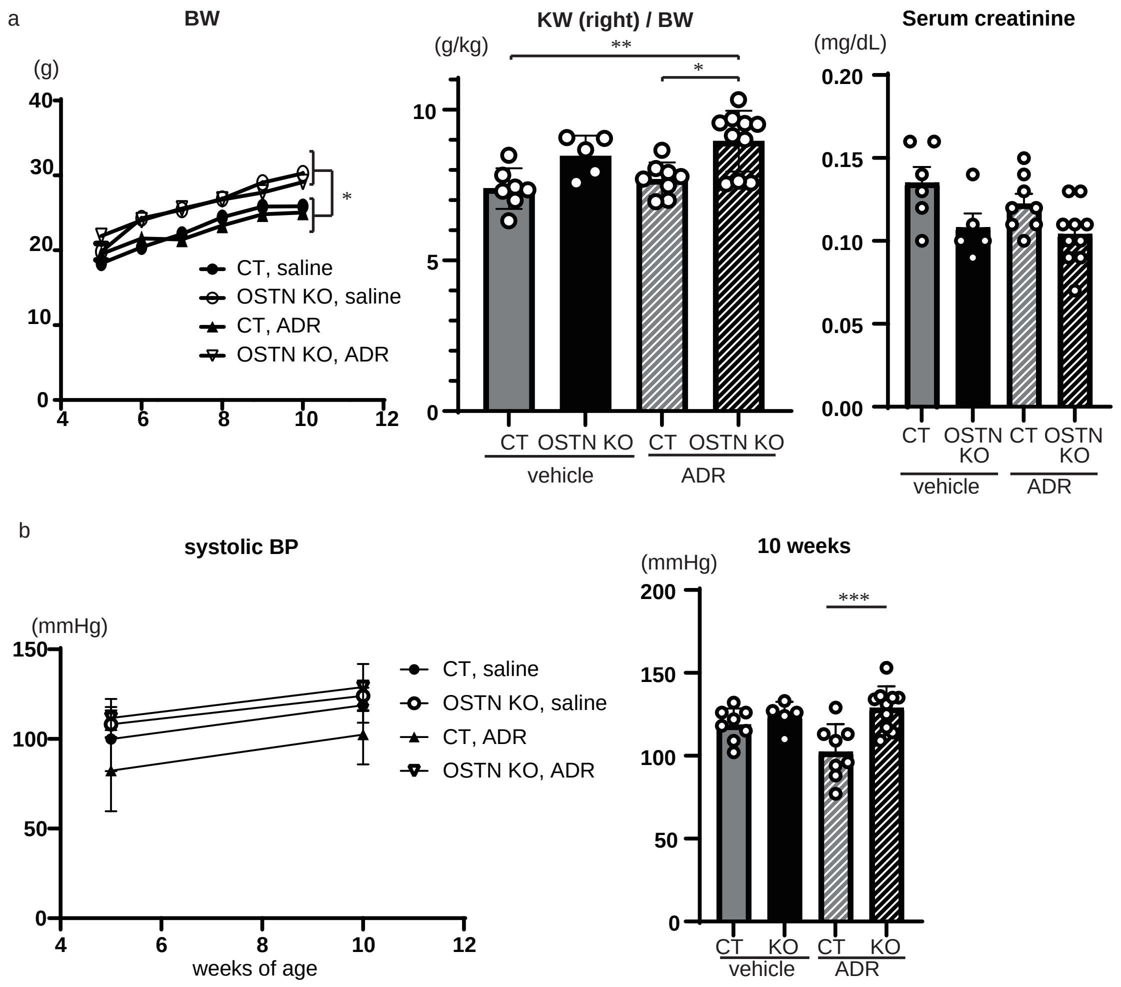

Supplementary Figure S3 (a) The BW at weeks 5, 6, 7, 8, 9 and 10 and right kidney weight, serum creatinine levels at 10 weeks of age (n = 5-10 per group). **P* < 0.05, CT + saline vs. OSTN KO + saline, or CT + ADR vs. OSTN KO + ADR by one-way ANOVA analysis.
(b) Systolic blood pressure at weeks 5 and 10 (n = 5-10 per group). ****P* < 0.001, KO + ADR vs. CT + ADR by one-way ANOVA analysis. :BW, body weight; CT, control mice; OSTN KO, OSTN KO mice; BP, blood pressure; Data are mean ± SD .

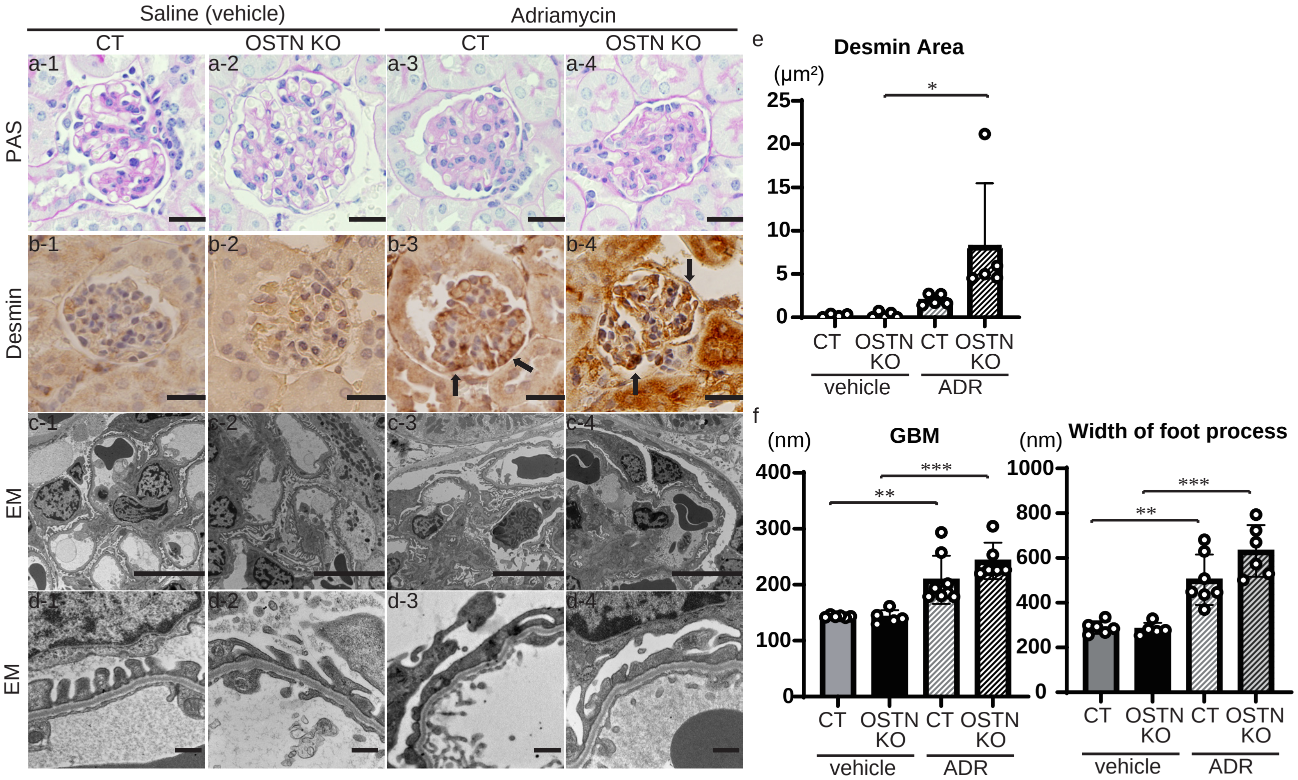

Supplementary Figure S4 (a) Light microscopic analysis stained with PAS. Bars, 20 μm. (b) Immunohistochemical study for desmin (magnification, ×400). Bars, 20 μm. (c, d) Electron microscopic analysis with lower magnification (c; magnification, ×2000, Bars, 10 μm.), and higher magnification (d; magnification, ×15000, Bars, 500 nm.). (e) Area of desmin-positive areas were analyzed.
(f) Thickness of glomerular basement membrane (GBM) and width of foot process effacements were measured. PAS, periodic acid-Schiff; EM, Electron microscopy; CT, control mice; OSTN KO, OSTN KO mice. Data are mean ± SD. *P < 0.05, **P < 0.01, ***P < 0.001 by one-way ANOVA analysis.

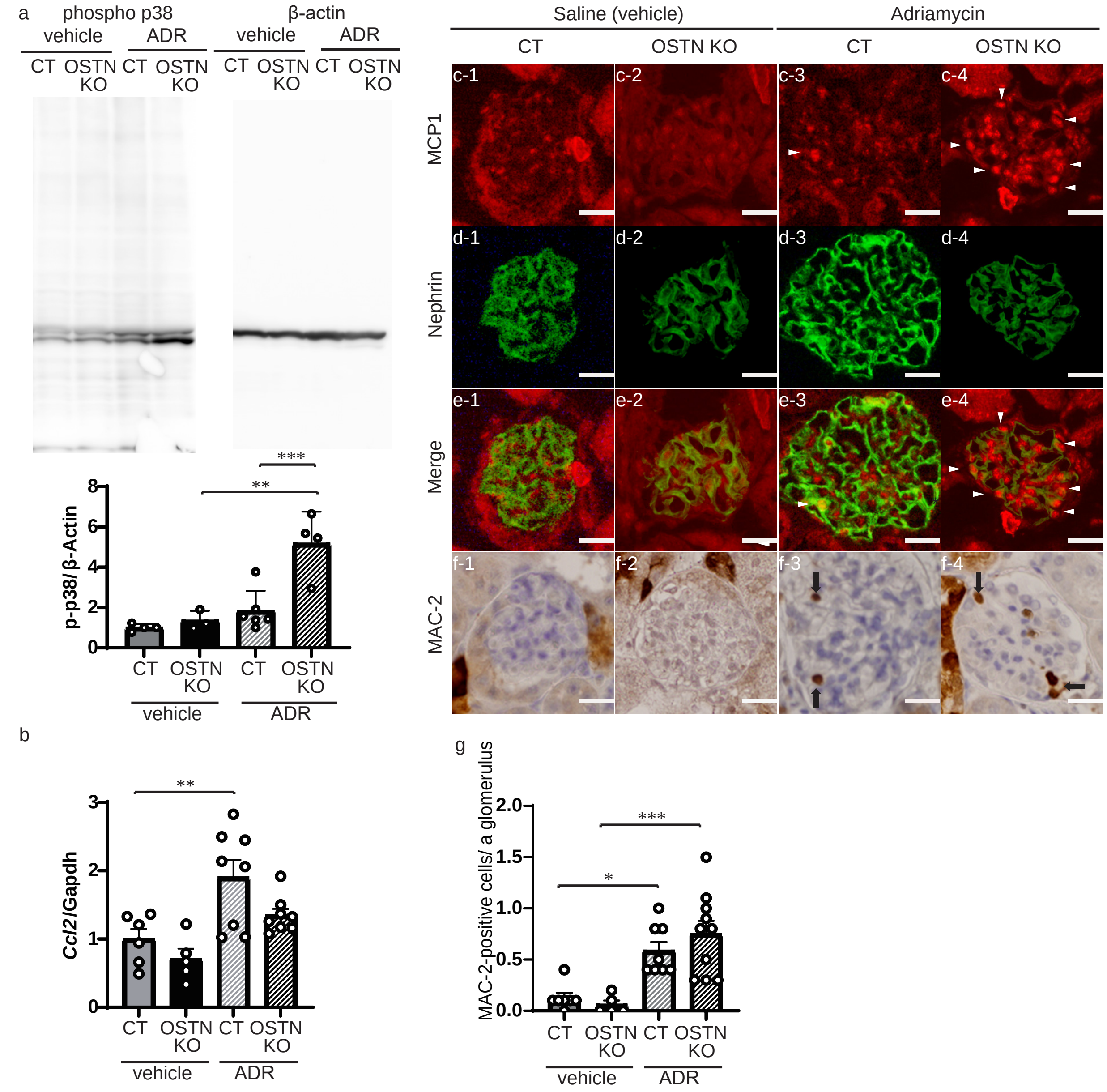

Supplementary Figure S5 (a) Glomerular p38 MAPK phosphorylation in four groups at 10 weeks of age.
The grouping of gels cropped from the same lines of the same gel. Full-length blots/gels are presented in Supplementary Figure S13d.
(b) Glomerular mRNA expression levels of *Ccl2* in four groups at 10 weeks of age. (c-e) Immunofluorescent studies for MCP1 (c; red) and nephrin (d; green) in four groups. The merged images are shown in e. (f, g) Immunohistochemical findings of MAC-2
(f), a macrophage marker, and the number of MAC-2-positive cells in glomeruli in four groups
(g). phospho p38, phosphorylated p38 MAPK; CT, control mice; OSTN KO, OSTN KO mice. Data are mean ± SD.
**P* < 0.05, ***P* < 0.01, ****P* < 0.001 by one-way ANOVA analysis.

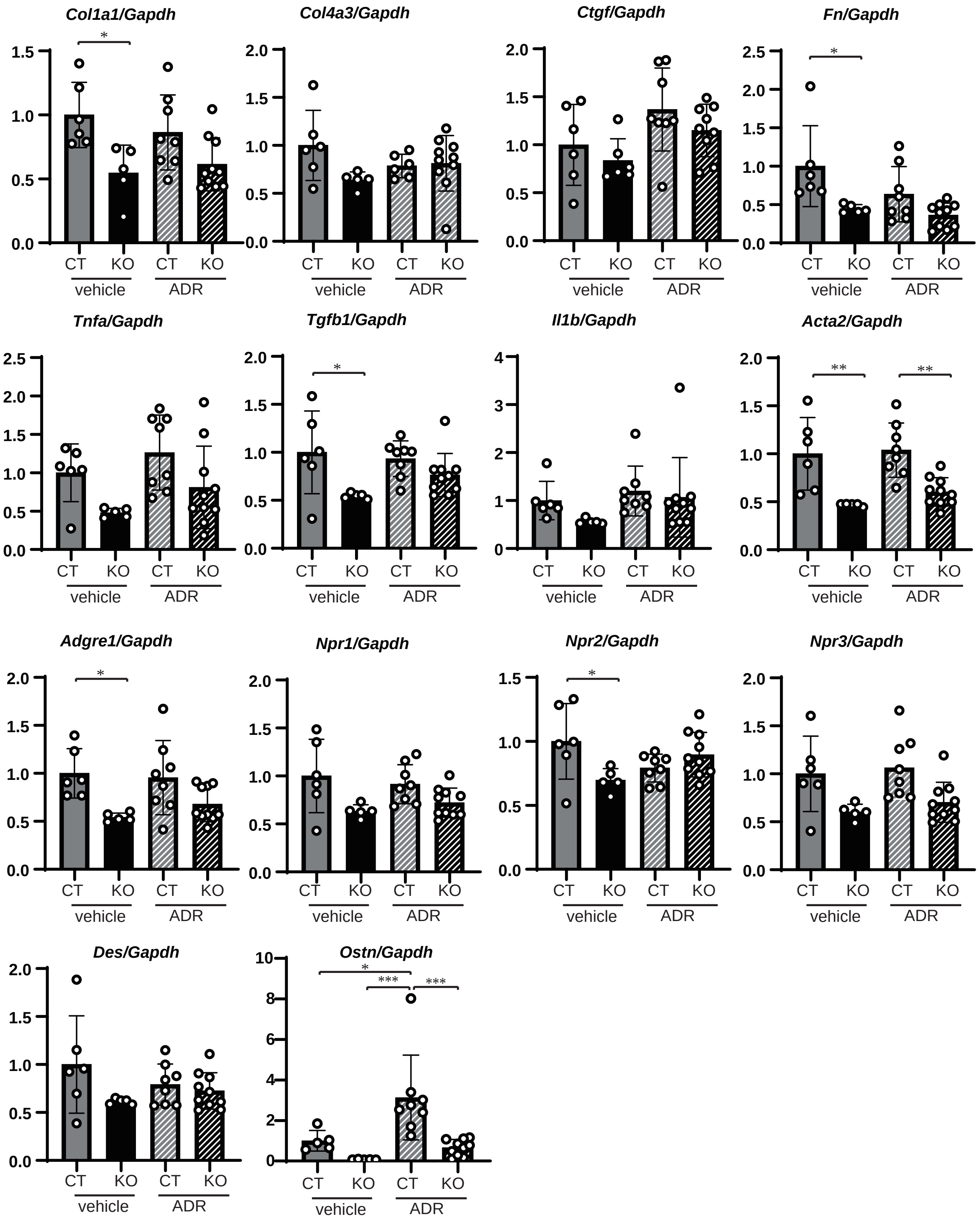

Supplementary Figure S6 The glomerular expression of *Col1a1* , *Col4a3* , *Ctgf* , *Fn* , *Tnfa* , *Tgfb1* , *Il1b* , *Acta2* , *Adgre1* , *Npr1* , *Npr2* , *Npr3* , *Des* and *Ostn* in OSTN-KO mice and control mice under ADR nephropathy. Data are mean ± SD **P* < 0.05, ***P* < 0.01, ****P* < 0.001 by one-way ANOVA analysis..

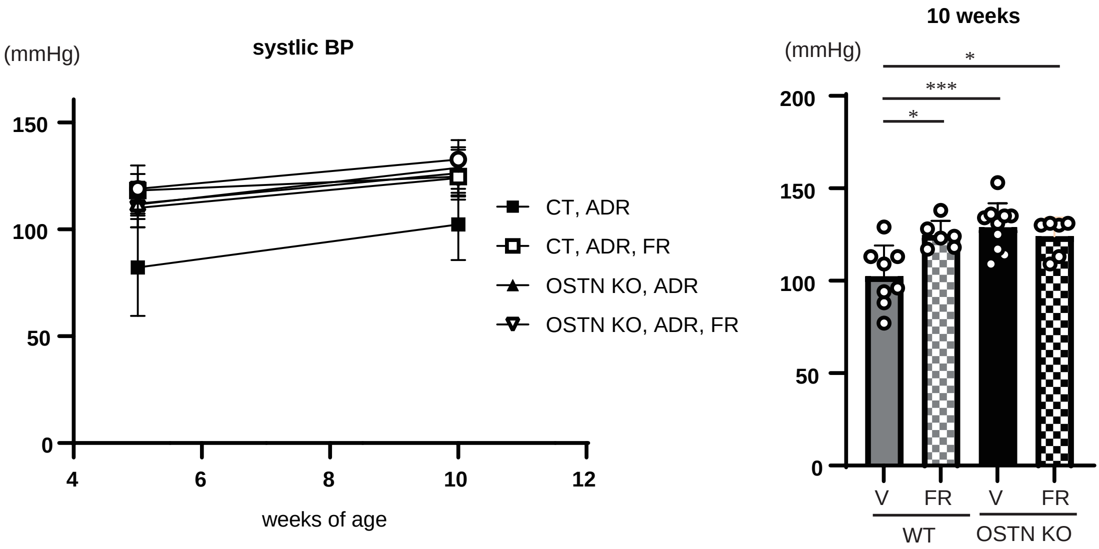

Supplementary Figure S7 Systolic blood pressure at weeks 5 and 10 (n = 6-10 per group).

BP, blood pressure; V, vehicle; FR, FR167653 CT, wild-type mice; OSTN KO, OSTN KO mice.

Data are mean ± SD. **P* < 0.05, ****P* < 0.001 by one-way ANOVA analysis.

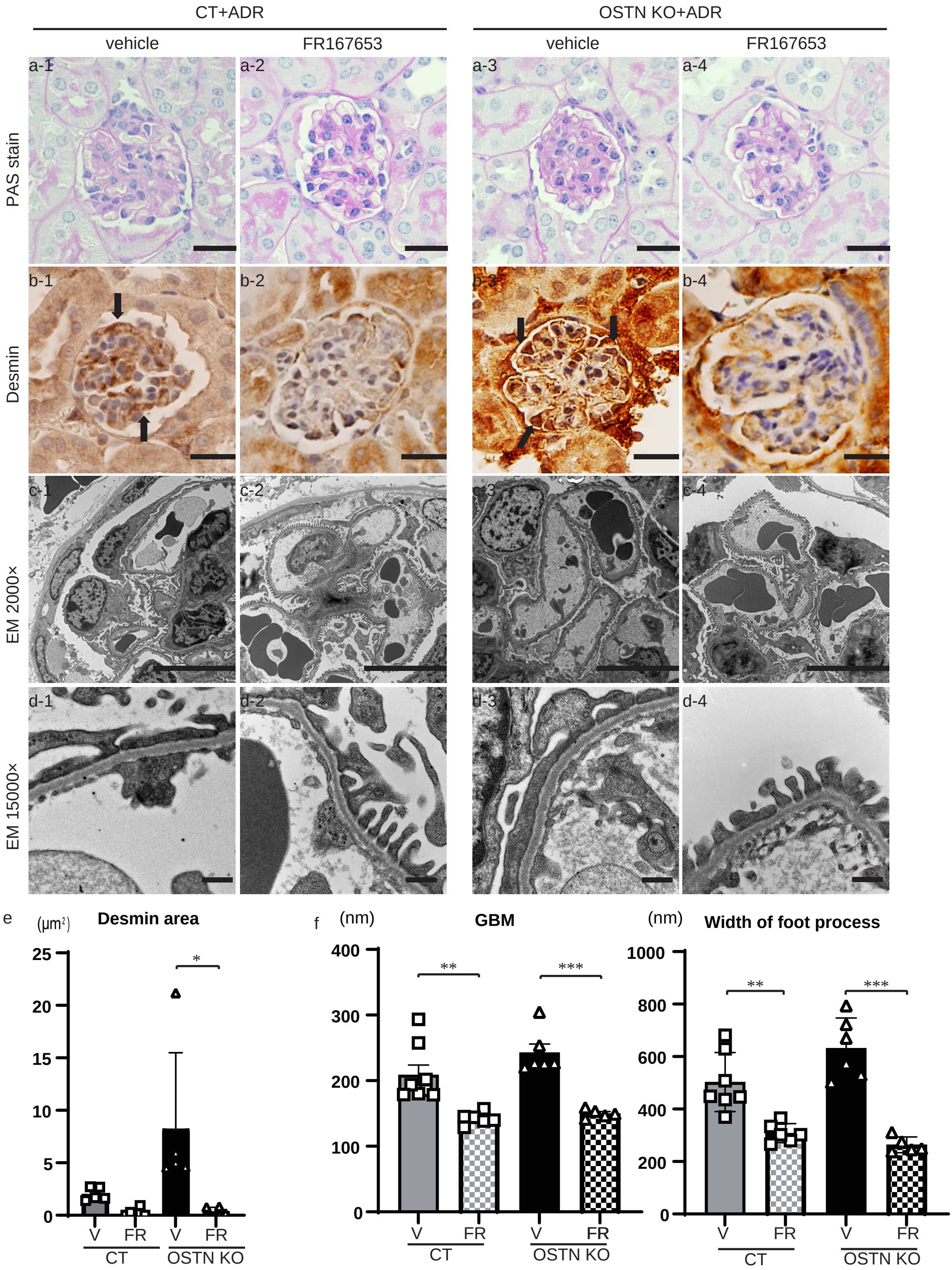

Supplementary Figure S8. (a) Light microscopic analysis stained with PAS. Bars, 20 μm. (b) Immunohistochemical study for desmin (magnification, ×400). Bars, 20 μm. (c,d) Electron microscopic analysis (c; magnification, ×2000, Bars, 10 μm)(d; magnification, ×15000, Bars, 500 nm). (e) Desmin-positive areas were analyzed. (f) Thickness of glomerular basement membrane and width of foot process effacements were measured. V, vehicle; FR, FR167653; CT, control mice; OSTN KO, OSTN KO mice. Data are mean ± SD. **P* < 0.05, ***P* < 0.01, ****P* < 0.001 by one-way ANOVA analysis.

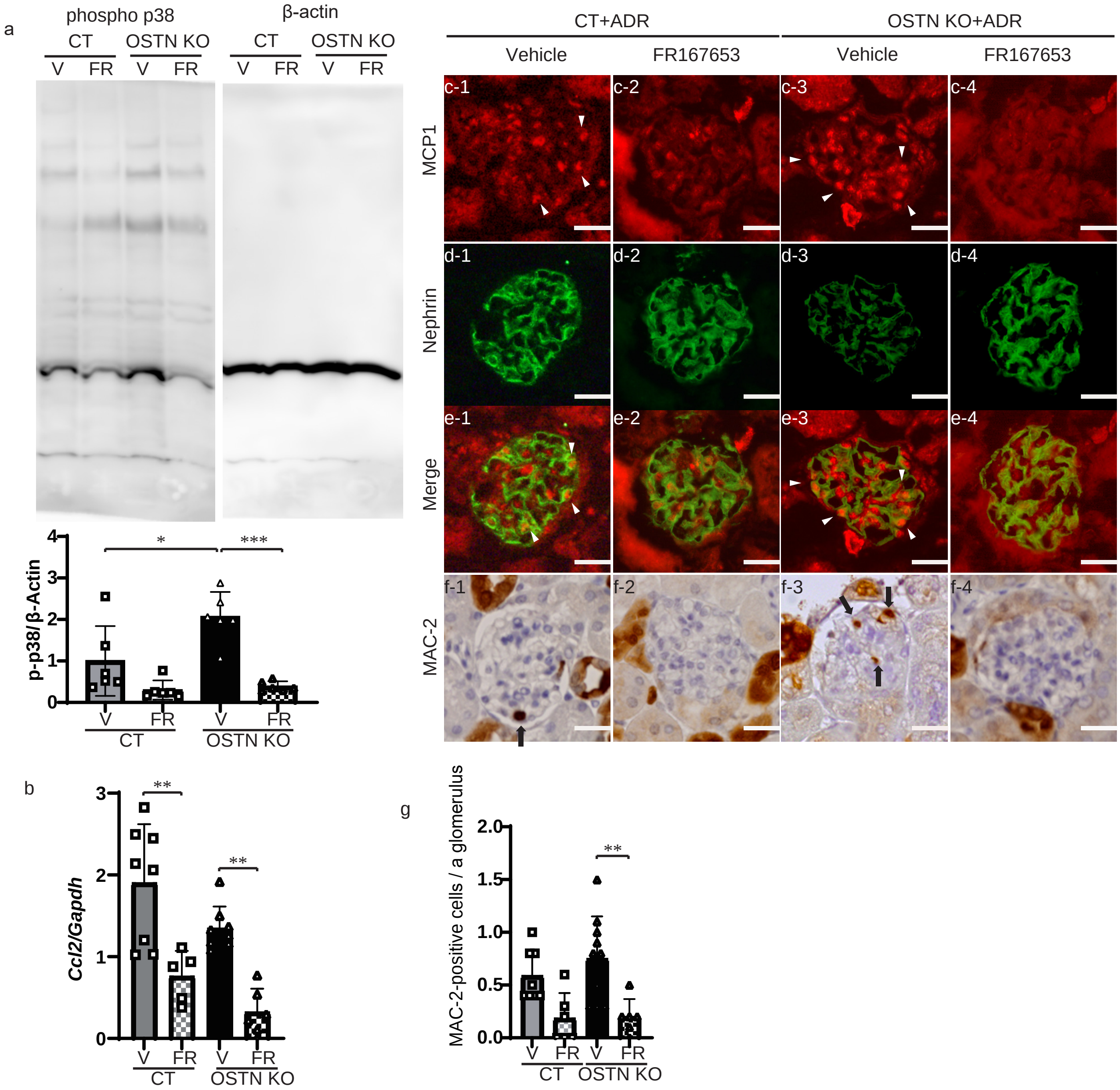

Supplementary Figure S9 (a) Glomerular p38 MAPK phosphorylation in four groups of mice at 10 weeks of age. The grouping of gels cropped from the same lines of the same gel after reblotting.Full-length blots/gels are presented in Supplementary Figure S13e. (b) Glomerular mRNA expression levels of *Ccl2* in four groups of mice at 10 weeks of age. (c-e) Immunofluorescent studies for MCP1 (c; red), nephrin (d; green), and merged images (e) in four groups. (f,g) Immunohistochemistry findings of MAC-2 (f) and the number of MAC-2-positive cells in glomeruli in four groups (g). phosphor p38, phosphorylated p38 MAPK; V, vehicle; FR, FR167653; CT, control mice; OSTN KO, OSTN KO mice. Data are mean ± SD. * $P < 0.05$, ** $P < 0.01$ by one-way ANOVA analysis.

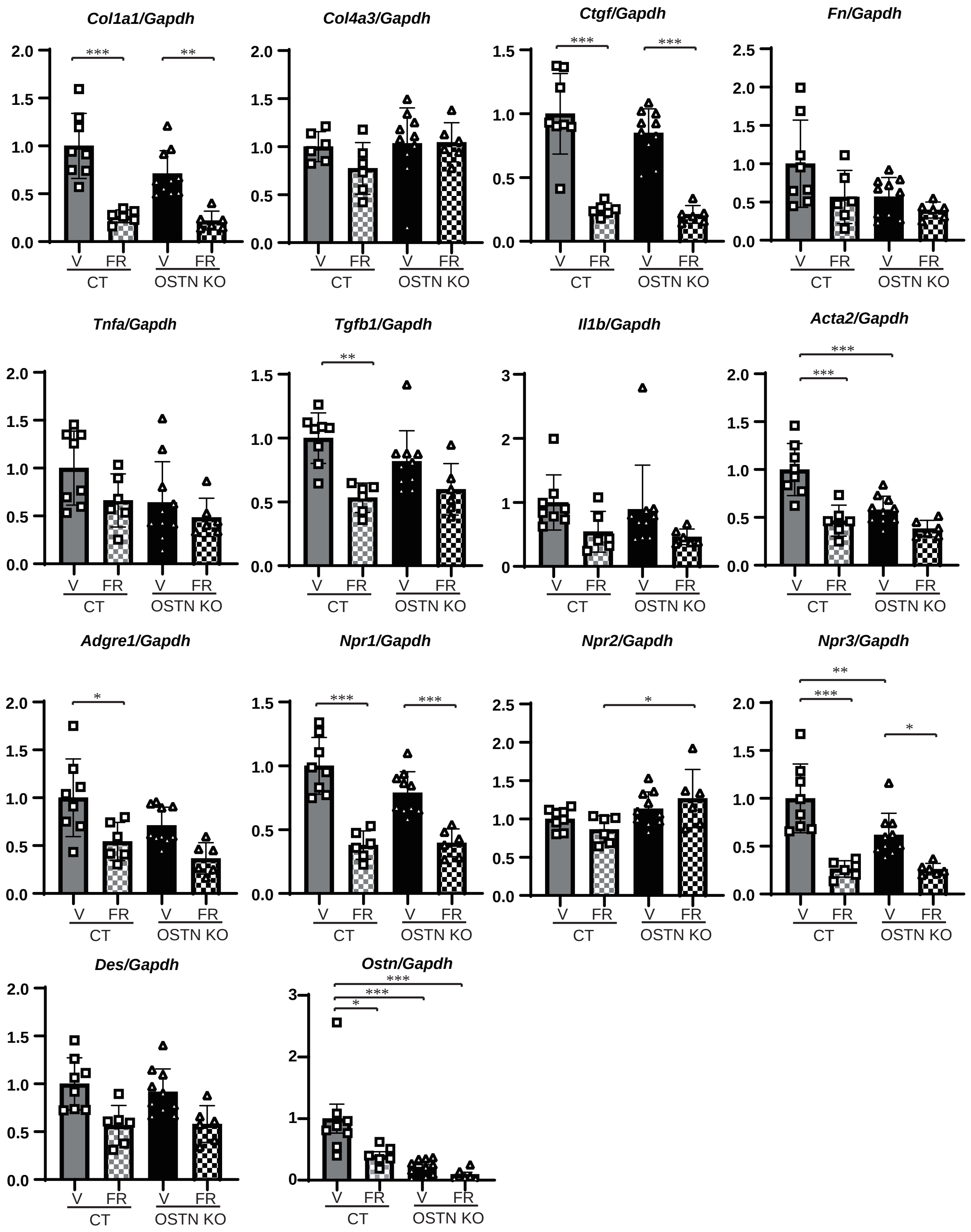

Supplementary Figure S10 The glomerular expression of *Col1a1* , *Col4a3* , *Ctgf* , *Fn* , *Tnfa* , *Tgfb1* , *Il1b* , *Acta2* , *Adgre1* , *Npr1* , *Npr2* , *Npr3* , *Des, Ostn* in FR167653-treated OSTN KO mice and control mice under ADR nephropathy. V, vehicle; FR, FR167653; CT, control mice; OSTN KO, OSTN KO mice. Data are mean ± SD. *P < 0.05, **P < 0.01, ***P < 0.001 by one-way ANOVA analysis

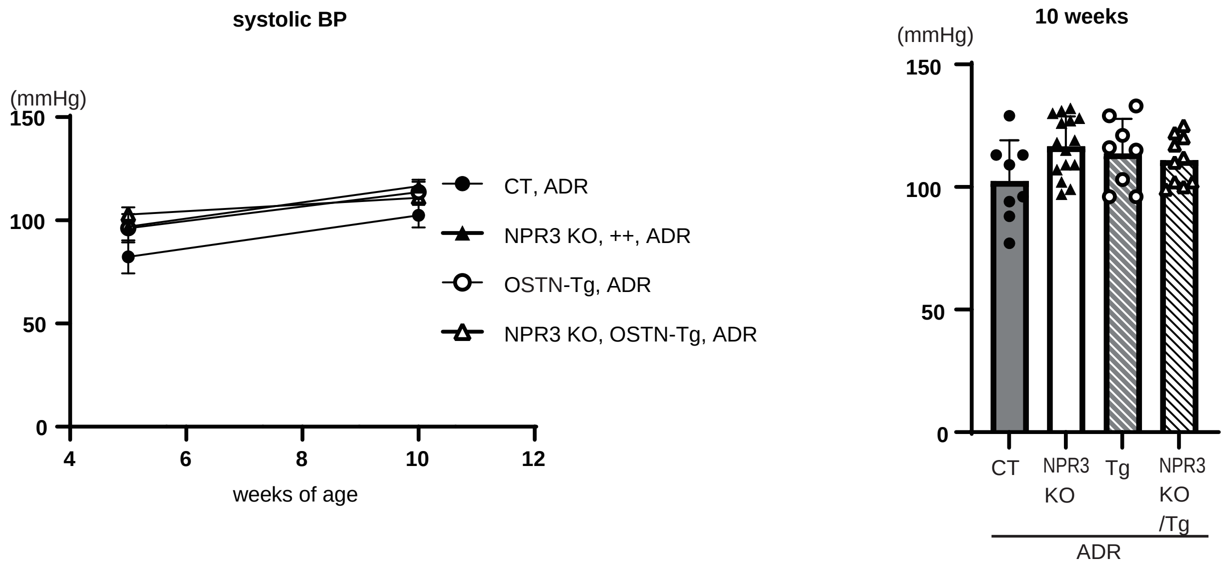

Supplementary Figure S11 Systolic blood pressure at weeks 5 and 10 (n = 8-15 per group). BP, blood pressure; CT, control mice; Tg, OSTN-Tg mice. Data are mean ± SD.

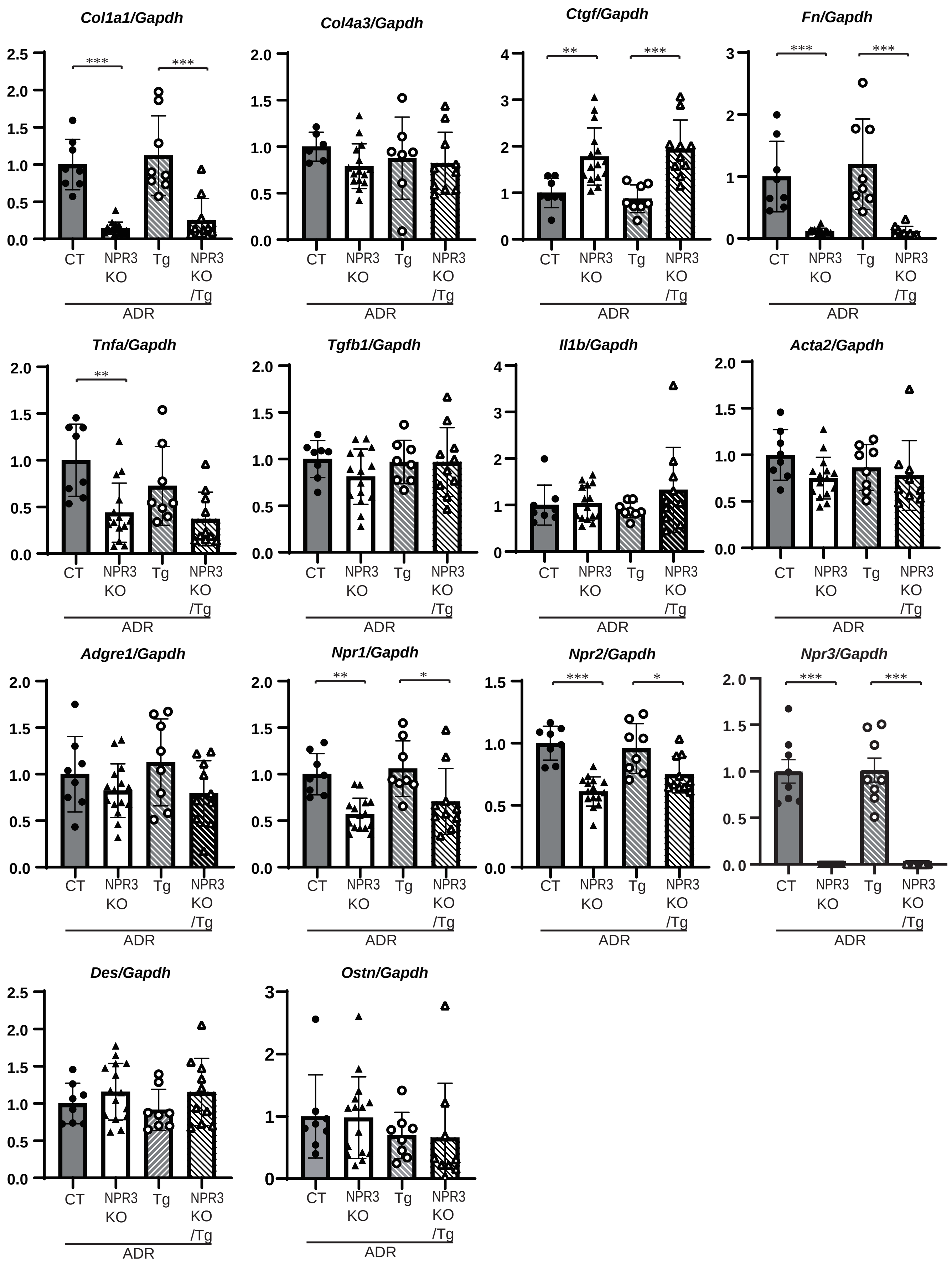

Supplementary Figure S12 The expression of *Col1a1* , *Col4a3* , *Ctgf* , *Fn* , *Tnfa* , *Tgfb1* , *Il1b* , *Acta2* , *Adgre1* , *Npr1* , *Npr2* , *Npr3* , *Des* and *Ostn* in glomeruli in NPR3 KO mice and OSTN-Tg mice under ADR nephropathy. CT, control mice; Tg, OSTN-Tg mice . Data are mean ± SD.
*P < 0.05, **P < 0.01, ***P < 0.001 by one-way ANOVA analysis

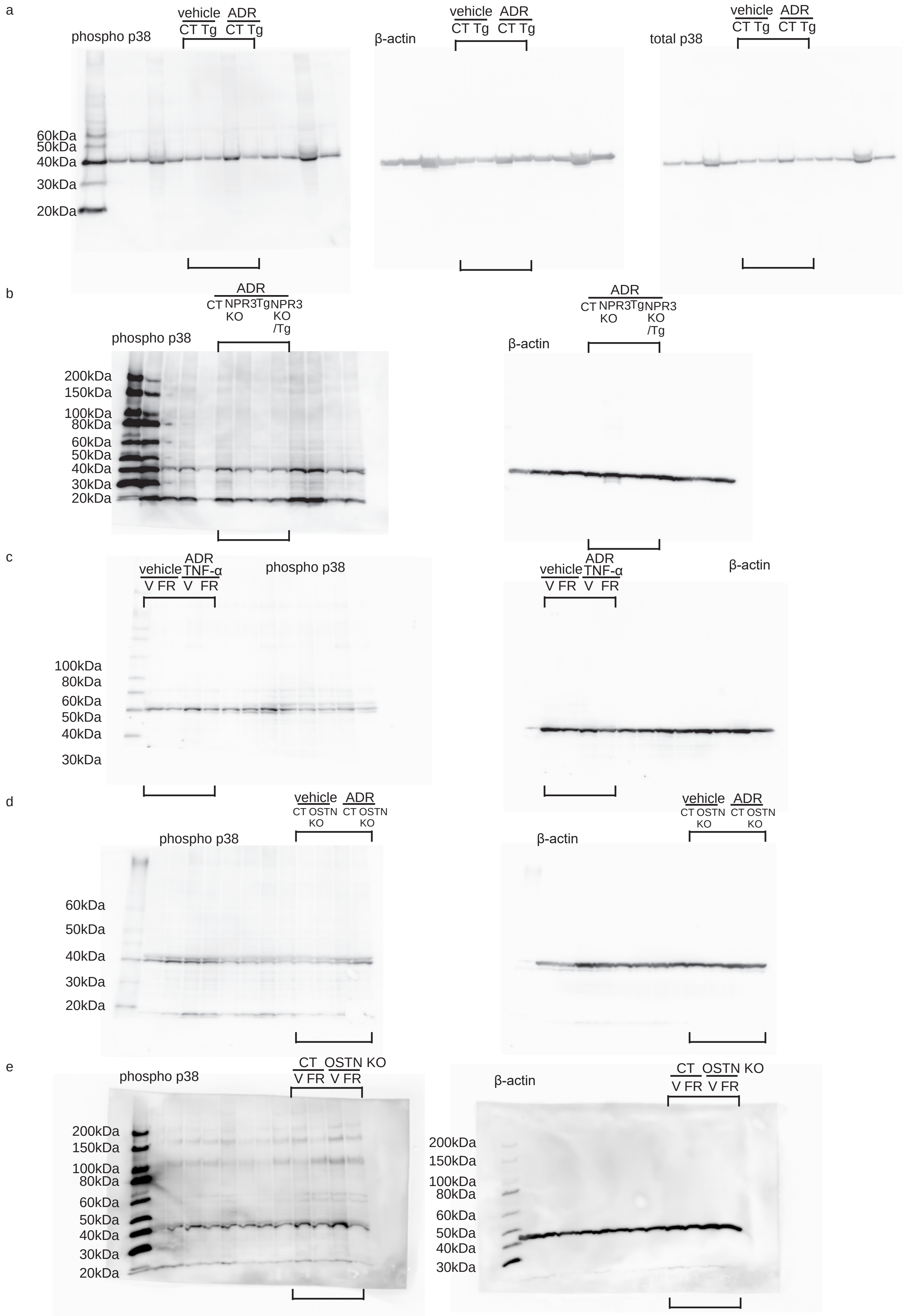

Supplementary Figure S13. Original whole images of the gels shown in Figure 3a, Figure 7a, Figure 8c and Supplementary Figure S5a, Supplementary Figure S9a.

(a) Original whole images of Figure 3a (b) Original whole images of Figure 7a. (c) Original whole images of Figure 8c (d) Original whole images of Supplementary Figure S5a. (e) Original whole images of Supplementary Figure S9a. Lanes shown by square brackets are cropped into each figure ; CT, control mice; Tg, OSTN-Tg mice, ADR, adriamycin, V, vehicle; A, 10^{-6} M ANP; OA, 10^{-6} M OSTN and 10^{-6} M ANP; FR, FR167653.

Table S1 TaqMan primer and probe sequcences

Target	Forward primer	Reverse primer	Probe
Ostn	5’-TCCCATGGATCGGATTGGTAGA-3’	5’-GTGCCATCTCACACAAGTAAGTC-3’	5’-FAM-CCATCAGCCTCTGGAACTGGAGAGCCG-TAMRA-3’
Tnfa	5’-CCTCTTCTCATTCCTGCTTGTG-3’	5’-GCCATTTGGGAACTTCTCATCC-3’	5’-FAM-CCACCACGCTCTTCTGTCTACTGAACTTCG-TAMRA-3’
Il1b	5’-CGGACCCCAAAAGATGAAGGG-3’	5’-CTGCCTGCCTGAAGCTCTTG-3’	5’-FAM-CAAACCTTTGACCTGGGCTGTCCTGATGAG-TAMRA-3’
Acta2	5’-CCTGACGCTGAAGTATCCGATAG-3’	5’-GGTGCCAGATCTTTTCCATGTC-3’	5’-FAM-ACACGGCATCATCACCAACTGGGA-TAMRA-3’
Npr2	5’-TCCACTTTCCACTCTGGCAATC-3’	5’-AGTTCTTCCCAGCGAATGCG-3’	5’-FAM-CCTGGGCACGGGAGTCACCTTCATCA-TAMRA-3’
Npr3	5’-CGGAATACTCGCACCTCACG-3’	5’-GCTGTAGACCAGGGCTGCA-3’	5’-FAM-CGCCTGCCTACGCCAAGATGGGAGA-TAMRA-3’
Des	5’-AGAGCAGGATCAACCTTC-3’	5’-CTGACAACCTCTCCATCC-3’	5’-FAM-CCTTCTCTGCTCTCAACTTCCG-TAMRA-3’
Ctgf	5'-GGTCAAATCCCTGTTGGTGAA-3'	5'-AAAGAAGCAGCAAGCACTTCCT-3'	5'-FAM-TTAAGAAATGGCTGGCTCAGGGTAAGGTCC-TAMRA-3'

Ostn , osteocrin; *Tnfa* , tumor necrosis factor-α; *Il1b* , interleukin-1β; *Acta2* , alpha smooth muscle actin; *Npr2* , natriuretic peptide receptor 2; *Npr3* , natriuretic peptide receptor 3; *Des* , desmin; *Ctgf* , connective tissue growth factor
